# Sex- and estrous-specific effects of paradoxical sleep deprivation: neurobehavioral changes and hippocampal neuroinflammation

**DOI:** 10.3389/frsle.2025.1611192

**Published:** 2025-09-22

**Authors:** Laura K. Olsen, Krysten A. Jones, Raquel J. Moore, Hunter McCubbins, Frances S. Curtner, Birendra Sharma, Candice N. Hatcher-Solis

**Affiliations:** ^1^Cognitive Neuroscience, 711^th^ Human Performance Wing, Air Force Research Laboratory, Wright-Patterson AFB, OH, United States; ^2^Oak Ridge Institute for Science and Education, Oak Ridge, TN, United States; ^3^Health and Performance Technologies Division, Blue Halo, Dayton, OH, United States; ^4^DCS Infoscitex, Dayton, OH, United States

**Keywords:** sleep deprivation, cognition, estrous cycle, microglia, hippocampus

## Abstract

With millions suffering from sleep disorders in today's society, a better understanding of sleep disruption related to cognitive outcomes is urgently needed. To that end, a preclinical investigation into the effects of paradoxical sleep deprivation (PSD) on neurobehavioral outcomes and associated hippocampal neuroinflammation was conducted in male and female rats. Due to epidemiological identification of sex differences in many aspects of sleep disorders, sex and estrous-cycle stage factors were investigated. Sprague-Dawley rats underwent 120 h of PSD using a modified multiple-platform “flowerpot” method. At 96 h of PSD, animals were trained on neurobehavioral Novel Object Recognition (NOR) and Passive Avoidance Task (PAT) paradigms. Before NOR/PAT testing, at 120 h PSD, the Elevated Zero Maze (EZM) was used to assess anxiolytic-like behavior. PSD-impaired PAT performance among males and females. In males after PSD, anxiolytic-like and locomotor behavior was increased, and NOR performance was impaired. Based on estrous cycle stages determined by cytological analysis of daily wet smears, females were found to exhibit estrous-specific differences across all neurobehavioral paradigms, with increased anxiolytic-like behavior and impaired PAT performance only among PSD females in estrus. Immunohistochemical analysis of the hippocampus after 120 h of PSD found microgliosis, but not astrogliosis, in the CA1/2 of males and females in estrus. This study contributes to a better understanding of the sex- and estrous-specific differences in sleep disruption–induced neurobehavioral outcomes and associated hippocampal inflammation. Further research is needed to investigate the molecular mechanisms underlying the interaction between estrous cycle, hippocampal microgliosis, and sleep-disrupted cognitive outcomes.

## 1 Introduction

With more than 50% of the U.S. population reporting sleep issues at some point in their lives and 40 million chronically suffering, sleep disorders have become an urgent concern in today's society (Léger et al., 2008; Li et al., 2011). Although sleep requirements change throughout life, at all ages, both the quality and quantity of sleep are important for health and cognitive function. Nearly all age groups in the United States have increased reporting of sleep disorders. Approximately 60%−75% of adolescents and 35% of adults report chronic insufficient sleep (Wheaton et al., 2018; Liu et al., 2016). Insufficient sleep is associated with overall cognitive impairment, which can increase the likelihood of accidents and injuries while operating machinery or motor vehicles, as well as medical errors and a loss in work productivity (Huyett and Bhattacharyya, 2021; Colten and Altevogt, 2006). Disrupted sleep quality/quantity has also been associated with multiple health conditions, including metabolic disorders, cardiovascular conditions, and neurological disorders (Gaultney, 2010; Owens and Adolescent Sleep Working Group, 2014; Lowry et al., 2012; Fitzgerald et al., 2011). Although the literature suggests that there are sex differences in the prevalence of sleep disorders and the susceptibility to subsequent disease risk associated with sleep disruption, inconsistent findings based on specific sleep dimension factors indicate that further research is needed to better understand the influence of sex on sleep disruption–related cognitive outcomes (Jono et al., 2022; Li et al., 2022; Lee et al., 2022).

The hippocampus mediates declarative learning and memory integration, encoding, and consolidation (Bendel et al., 2005; Bird and Burgess, 2008). The activation of neural processes within the hippocampus leads to molecular and structural synaptic plasticity changes underlying these cognitive phenomena. Although the behavioral significance is not fully understood, sex-specific differences in dendritic spine densities and structures of pyramidal neurons of the hippocampus suggest that sex-specific differences in synaptic plasticity may exist (Parducz and Garcia-Segura, 1993; Komatsuzaki et al., 2005). As regulators of the brain's microenvironment, glial cells within the hippocampus modulate neuronal synaptic plasticity (Miyamoto et al., 2016; Schafer et al., 2012). Microglia, the resident immune cells of the brain, can participate in healthy synaptic regulation or runaway pro-inflammatory response depending on their activation state (Schafer et al., 2012; Bardou et al., 2014). Astroglia crosstalk with microglia during inflammatory insults also mediates the progression of neuroinflammation within the brain. Activated microglia and astroglia release pro-inflammatory mediators that modulate synaptic protein levels and dendritic spine densities (Prada et al., 2018). Within the hippocampus, these glial cells influence learning and memory-associated activity-dependent synaptic plasticity pathways (Golia et al., 2019). Increases in activated microglia and pro-inflammatory cytokines have been found after chronic sleep deprivation (Wadhwa et al., 2019). Although the current understanding from the literature identifies the pro-inflammatory glial response in the hippocampus as mediating, in part, cognitive impairment during disrupted sleep, the effect of sex has not yet been investigated.

First, this study sought to characterize the cognitive behavioral outcomes of paradoxical sleep deprivation (PSD) across a battery of neurobehavioral tests in male and female rodents. Female animals have historically been generally excluded from neurobehavioral investigations due to the “confounding” variable of the estrous cycle (Beery and Zucker, 2011). In this study, females' estrous cycle was tracked to determine the influence of female sex hormones on cognitive behavioral outcomes of PSD. Finally, this study investigated glial cell expression in hippocampal subregions after PSD in male and female rodents to determine if there are sex- or estrous-specific effects.

## 2 Materials and methods

### 2.1 Animals

All animal activities were conducted in an Association for Assessment and Accreditation of Laboratory Animal Care (AAALAC) International–accredited facility in compliance with all federal regulations governing the protection of research and animals and Department of Defense Instruction 3216.01. This study was approved by the Wright-Patterson Air Force Base Institutional Animal Care and Use Committee (IACUC). Male or female Sprague-Dawley rats (Charles River) were group-housed by biological sex with *ad libitum* access to water and food on a 12-h light cycle. Rats were acclimated to the facility for at least 1 week before the study began and were assigned to the control or PSD groups. Rats underwent a total of 120 h of PSD during behavioral testing (Supplementary Figure S1A). At the time of behavior, all rats were aged 10–12 weeks. The male and female cohorts were not run in parallel (males first), but the same exact experimental conditions were utilized for PSD, neurobehavior, and tissue collection, including Zeitgeber time to avoid circadian rhythm effects. In female rates, estrous cycle was tracked by wet smear collection for at least 5 days during experimentation.

### 2.2 PSD

A modified “flowerpot” method using multiple platforms in a large container was utilized to allow animals to undergo PSD in a group-housing environment and prevent stress induced by immobilization on a single platform (Coenen and Van Luijtelaar, 1985). Animals were placed on small circular platforms (5 cm in diameter) spaced 8 cm apart and ~3 cm above the water level. In this condition, animals are able to rest on the small platform, but if they have sleep-related muscle tone loss, they are awakened by falling into the water below. To prevent hypothermia stress, the water was warmed to 26–28 °C, and the ambient room temperature was approximately 80 °C. Due to the reliance on loss of muscle tone to enforce wakefulness, this method is considered a model of paradoxical or REM sleep deprivation (Vollert et al., 2011). Control animals were placed on large square platforms (14 cm × 14 cm) that allowed for normal sleeping behavior above the water. Food and water were provided *ad libitum*. The water containers were cleaned daily, during which time animals were placed in dry cages for 30–60 min and kept awake with grooming and exploratory behavior. No recovery sleep was allowed prior to neurobehavioral assessment or tissue collection.

### 2.3 Wet smears

The female Sprague-Dawley rats' estrous-cycle stage was tracked by analyzing wet smear collections. Vaginal lavage was collected three times with a pipette and the same 10 μl of sterile saline. Samples were air-dried on slides, and hematoxylin and eosin staining was performed using a Tissue-Tek Prisma Plus Automated Slide Stainer. The slides were first immersed in hematoxylin (7017, Astral Diagnostic) and then eosin (s176, Poly Scientific R&D Corp). Next, a series of washes in denatured alcohol (HC-1100, Fisherbrand) and xylene (HC700, Fisherbrand) were completed. The stained samples were evaluated to determine cell cytology. The estrous cycle stage was classified by the ratios of leukocytes, nucleated epithelial cells, and cornified epithelial cells. Estrous cycle stage was assigned from wet smears collected on training day for cognitive neurobehavioral paradigms and immunohistochemical analyses. To analyze the effects of estrogen, female rats in metestrus and diestrus cycle stages were grouped as “diestrus phase,” while those in proestrus and estrus cycle stages were grouped as “estrus phase”.

### 2.4 Elevated Zero Maze

The Elevated Zero Maze (EZM) test was performed to assess anxiety-like behaviors (Supplementary Figure S1B). The EZM was elevated 65 cm above the floor, and the circular platform (outer diameter 100 cm) was divided into four equal arms. Two opposite arms were open, and the remaining two arms were closed (surrounded by 30-cm-high dark walls). The open-arm regions were surrounded by a 1-cm-high edge. Animals were placed in the middle of a closed arm (left or right, randomized for each trial) and allowed to explore for 5 min. During the trial, the rat's activity was monitored using video-tracking software, Ethovision XT (v11.5, Noldus). EZM data analysis was performed for all males (control *n* = 12, PSD *n* = 9), all females (control *n* = 19, PSD *n* = 17), diestrus females (control *n* = 14, PSD *n* = 13), and estrus females (control *n* = 5, PSD *n* = 4).

### 2.5 Novel Object Recognition

The Novel Object Recognition (NOR) paradigm was used to investigate changes in recognition memory (Supplementary Figure S1C). Rats were allowed to explore the NOR arena (60 cm × 60 cm × 38 cm) in the presence of two similar objects (~8 cm radius, approximately 6-cm-tall crown-shaped glass item spray-painted black) for 3 min. The following day, the rats were again allowed to explore the NOR arena in the presence of the same object and a novel object (approximately 8 cm radius, approximately 10-cm-tall rook-shaped glass item spray-painted black) for 3 min. The objects were 47 cm apart on opposite sides of the arena. The rats were habituated to the arena for 2 min before the object exposure on training and testing days. The Ethovision XT video-tracking software was used to monitor object exploration activity. The NOR data analysis was performed for all males (control *n* = 9, PSD *n* = 6), all females (control *n* = 18, PSD *n* = 11), diestrus females (control *n* = 6, PSD *n* = 7), and estrus females (control *n* = 12, PSD *n* = 4). Novel object preference (NP) was calculated on testing day using the following equation:


$$\begin{array}{l} \text{NP}=\frac{\left(Nnovel\;-Nfamiliar\right)}{\left(Nnovel+Nfamiliar\;\right)}. \end{array}$$

### 2.6 Passive Avoidance Task

The Passive Avoidance Task (PAT) was used to detect changes in associative aversion learning (Supplementary Figure S1D). On habituation day, rats were allowed to explore the lit chamber and dark chamber (25 cm × 21 cm × 17 cm) of the apparatus (Gemini Avoidance System, San Diego Instruments Inc.) for 5 min. After approximately 24 h, the rats were placed in the lit chamber and again allowed to enter the dark chamber. If the rat chose to cross to the dark chamber, the gate separating the chambers closed, and a one-time foot shock (0.75 mA) was delivered in the dark chamber. After the foot shock, the rat remained in the dark chamber for an additional 30 s to facilitate aversive training. The following day, the rat was returned to the lit chamber. Latency to enter the dark chamber from the lit chamber was measured and analyzed to determine associative learning retention. PAT data analysis was performed for all males (control *n* = 12, PSD *n* = 9), all females (control *n* = 20, PSD *n* = 20), diestrus females (control *n* = 7, PSD *n* = 11), and estrus females (control *n* = 13, PSD *n* = 9).

### 2.7 Immunohistochemistry staining

After completion of behavioral paradigms on testing day, rats were euthanized under deep anesthesia by exsanguination with saline perfusion. The brain was immediately extracted and fixed in 4% paraformaldehyde for 48 h. Fixed brain tissue was then transferred to 30% sucrose for at least 3 days. A sliding microtome (SM2010R, Leica) was used to collect 30-μm coronal hippocampal slices for immunostaining of ionized calcium binding adaptor molecule (Iba1) or glial fibrillary acidic protein (GFAP). Tissue was blocked [phosphate buffered saline (PBS) with 2% goat serum, 0.2% Triton X-100] at room temperature for 1 h and incubated in rabbit anti-Iba1 (1:500, Wako, 019-19741) or rabbit anti-GFAP (1:2000, Agilent, Z033429-2) overnight at 4 °C. The following day, mouse anti-NeuN (1:2500, Sigma, MAB377) was added to the primary antibody incubations for 2 h at room temperature. The tissues were then rinsed five times (PBS, 5 min/rinse) before the slices were incubated in secondary antibodies (goat anti-rabbit Alexa Fluor 488 and goat anti-mouse Alexa Fluor 594, Jackson ImmunoResearch, 111-545-144 and 115-585-146) for 1 h at room temperature. The tissues were rinsed (PBS, 5 min/rinse, 5 rinses) and then mounted using Fluroshield with DAPI (Sigma, F6057). Fluorescent images were collected using an Olympus microscope at 20× magnification and quantified using QuPath and ImageJ. NeuN was used to identify the stratum radiatum (SR) and pyramidal layer (PL) of Cornu Ammonis (CA) CA1 and CA2. The quantification of Iba1- or GFAP-positive cells in each region was normalized to DAPI counts. Immunohistochemistry (IHC) quantification was performed on three technical replicates for each rat biological sample by a blinded experimenter. Representative Iba1 and GFAP images for all hippocampal regions are provided in in the Supplementary Figures S4, S7. Iba1 data analysis was performed for all males (control *n* = 5, CA1 PSD *n* = 6, CA2 PSD *n* = 5), all females (control, *n* = 8; PSD, *n* = 9), diestrus females (control *n* = 4, PSD *n* = 5), estrus females (control *n* = 4, PSD *n* = 4). GFAP data analysis was performed for all males (CA1 control *n* = 3, CA2 control *n* = 4, PSD *n* = 6), all females (control *n* = 8, PSD *n* = 8), diestrus females (control *n* = 4, PSD *n* = 4), estrus females (control *n* = 4, PSD *n* = 4).

### 2.8 Statistical analysis

All data were examined for normality and homogeneity of variance using the Shapiro–Wilk and Levene's tests, respectively. Statistical significance of the behavioral data and IHC expression data sets was determined by unpaired two-tailed *t*-test, the Mann–Whitney *U* test, or two-way between-group analysis of variance (ANOVA). The *post hoc* analysis was performed by Bonferroni comparison. Data are presented as means ± the standard error of the mean. A calculated *p* < 0.05 was considered statistically significant. Detailed statistical analysis results are provided in Supplementary Tables S1–S9.

## 3 Results

### 3.1 PSD effects on anxiety measures

**Figure 1:**
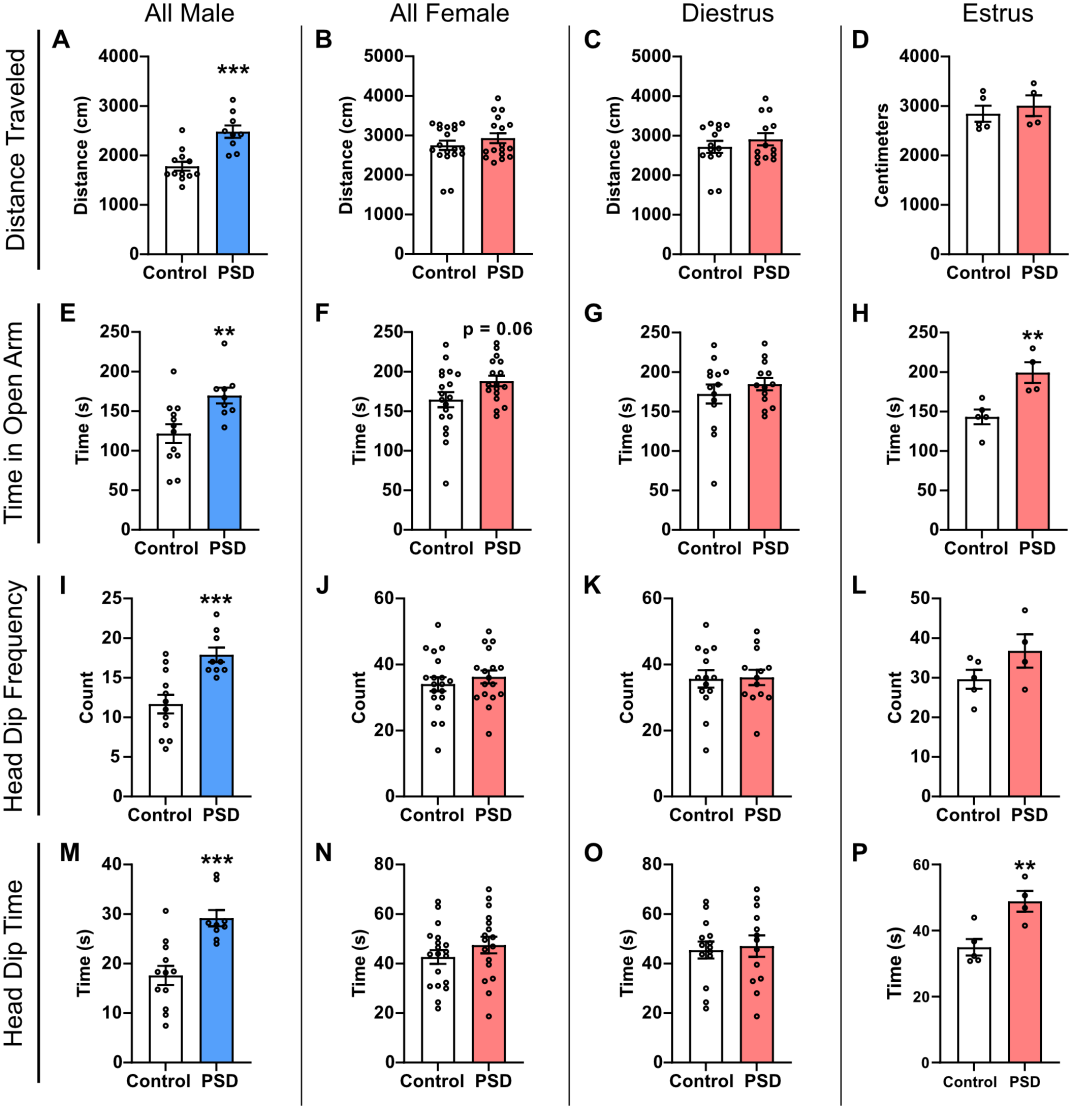
Paradoxical sleep deprivation (PSD) effects on anxiety-like behavior in the Elevated Zero Maze (EZM) paradigm are sex-dependent. **(A)** Distance traveled in the EZM was increased for male PSD rats. **(B)** PSD did not affect distance traveled in all female subjects or for females in **(C)** diestrus or **(D)** estrus. **(E)** In all male subjects, PSD decreased anxiety-like behaviors as indicated by an increase in the time spent in the EZM open arm. **(F)** An increase in time spent in the open arm approached significance for all female PSD subjects. **(G)** PSD before EZM did not influence time spent in the open arm for females in diestrus, and **(H)** increased time spent in the open arm for PSD females in estrus. **(I)** Head dip frequency was increased for PSD male rats. **(J)** Head dip frequency was not affected by PSD for all female subjects, including females in **(K)** diestrus and **(L)** estrus. **(M)** Head dip time was increased for PSD male rats. **(N)** Head dip time was not affected by PSD for all female rats or **(O)** females in diestrus. **(P)** Head dip time was increased for PSD estrus females. Bar graph data are presented as mean ± standard error of the mean (SEM) for all males (control *n* = 12, PSD *n* = 9), all females (control *n* = 19, PSD *n* = 17), diestrus females (control *n* = 14, PSD *n* = 13), and estrus females (control *n* = 5, PSD *n* = 4). See Supplementary Table S1 for full statistical results. **p < 0.01 vs. control. ***p < 0.001 vs control.

The EZM paradigm was used to examine the impact of paradoxical PSD on anxiety-like behaviors in male and female rats. After 120 h of PSD, general locomotion was statistically greater among PSD male rats compared to their control group (Figure 1A). No difference in distance traveled was observed among female rats, regardless of estrous cycle stage (Figures 1B–D). Compared to control rats, male PSD rats spent more time in the open arm, which is often thought to provoke anxiety (Figure 1E). Time in the open arm also approached significance between all female PSD and controls (Figure 1F). Among the female rats, when the estrous cycle was examined, a significant increase in the amount of time in the open arm was only found for PSD females in estrus (Figures 1G, H).

Head dip activity was also assessed as an additional measure of relative anxiety. Head dip frequency was significantly different between control and PSD groups for all males, with the PSD rats displaying more head dips than controls (Figure 1I). There was no significant difference in head dip frequency between the PSD and control group among all female rats (Figure 1J). Estrous cycle stage did not significantly influence head dip frequency between control and PSD females, although there was an increasing trend among PSD rats in estrus (Figures 1K, L). Similar to head dip frequency, the total head dip time was significantly increased for PSD male rats compared to the male control (Figure 1M). Head dip time was also estrous cycle–dependent, with only PSD females in estrus at the time of testing having a significant increase as compared to the control group (Figures 1N–P). Collectively, these results indicate that PSD reduces anxiety-like behaviors in male and female rats. This effect is estrous cycle–dependent in female rats because significant differences in anxiety behavior are only observed in the estrus phase. However, this effect is an interaction with PDS because there was no estrous cycle–dependent effect in the control condition across all EZM measures (Supplementary Figures S2A–D).

### 3.2 PSD effects on memory and learning

**Figure 2:**
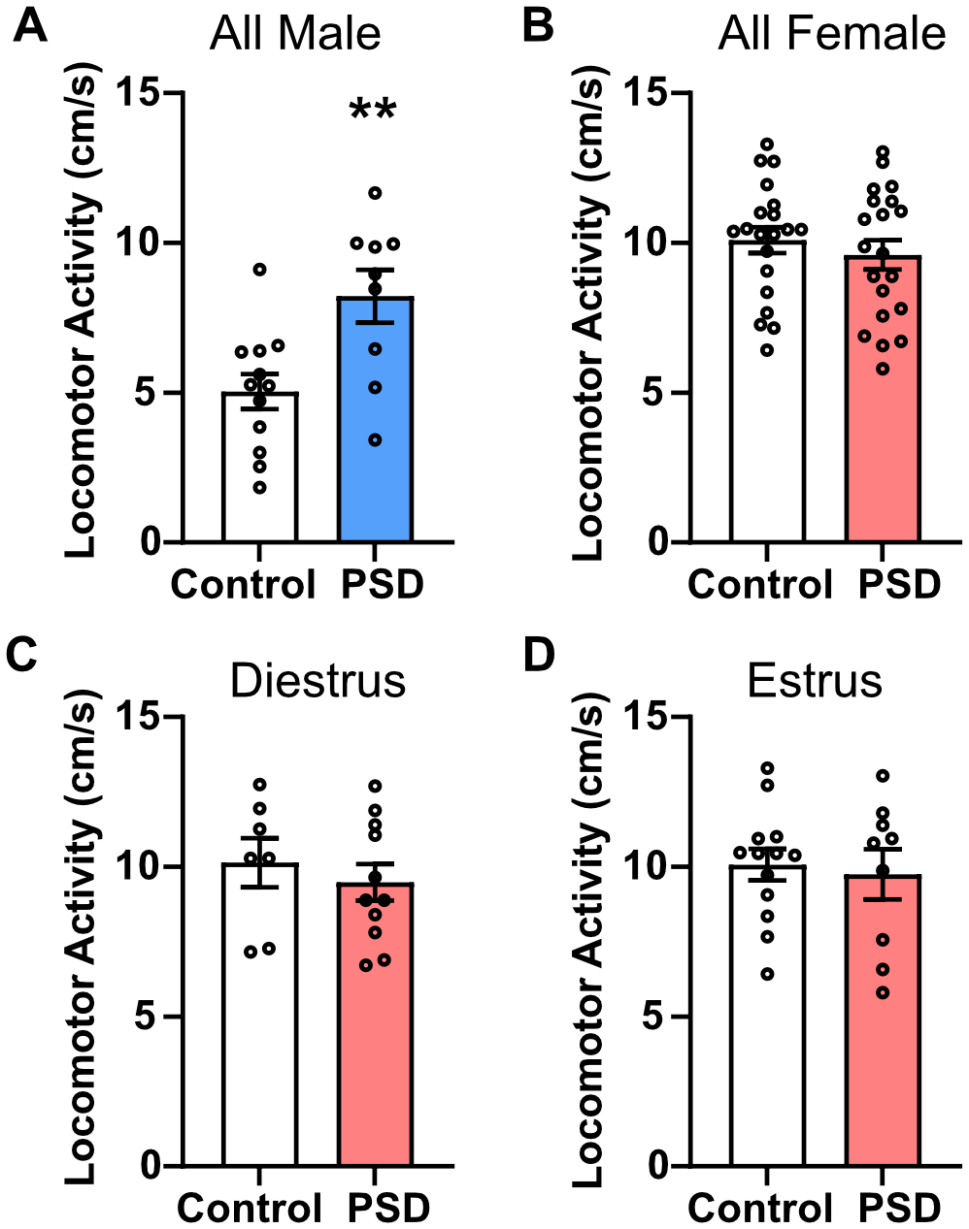
Locomotor activity is only increased by paradoxical sleep deprivation (PSD) in male rats. **(A)** Locomotor activity was increased for male PSD rats compared to controls. **(B)** PSD did not affect locomotor activity in all female subjects or for females in **(C)** diestrus or **(D)** estrus. Bar graph data are presented as mean ± standard error of the mean (SEM) for all males (control *n* = 12, PSD *n* = 9), all females (control *n* = 20, PSD *n* = 20), diestrus females (control *n* = 7, PSD *n* = 11), and estrus females (control *n* = 13, PSD *n* = 9). See Supplementary Table S2 for full statistical results. **p < 0.01 vs. control.

The NOR and PAT paradigms were used to examine the effects of paradoxical PSD on recognition memory and learning/memory, respectively. Rats were habituated to the NOR arenas 24 h before training sessions, at which point they were under 72 h of PSD. After 96 h of PSD, the NOR and PAT training sessions were completed. The NOR and PAT testing were completed the following day after an additional 24 h of PSD. During the NOR testing session, general locomotor activity was also measured before the introduction of the familiar and novel objects. Male PSD rats had significantly greater locomotor activity than male controls (Figure 2A). No significant difference between the control and PSD groups was detected among all females or based on estrous cycle phase (Figures 2B–D). These results are consistent with the level of locomotor activity observed by distance traveled in EZM. General locomotor activity earlier in the PSD paradigm was determined by examining velocity during the 5-min habituation period, which occurred after 72 h of PSD. Similar to general locomotor activity during the testing session after 120 h of PSD, male PSD rats exhibited greater locomotor activity compared to male controls (Supplementary Figure S3A). There were no significant group differences in locomotor activity among the female rats (Supplementary Figures S3B–D). However, female PSD rats in estrus had a decreasing trend in locomotor activity compared to their corresponding control group (Supplementary Figure S3D).

**Figure 3:**
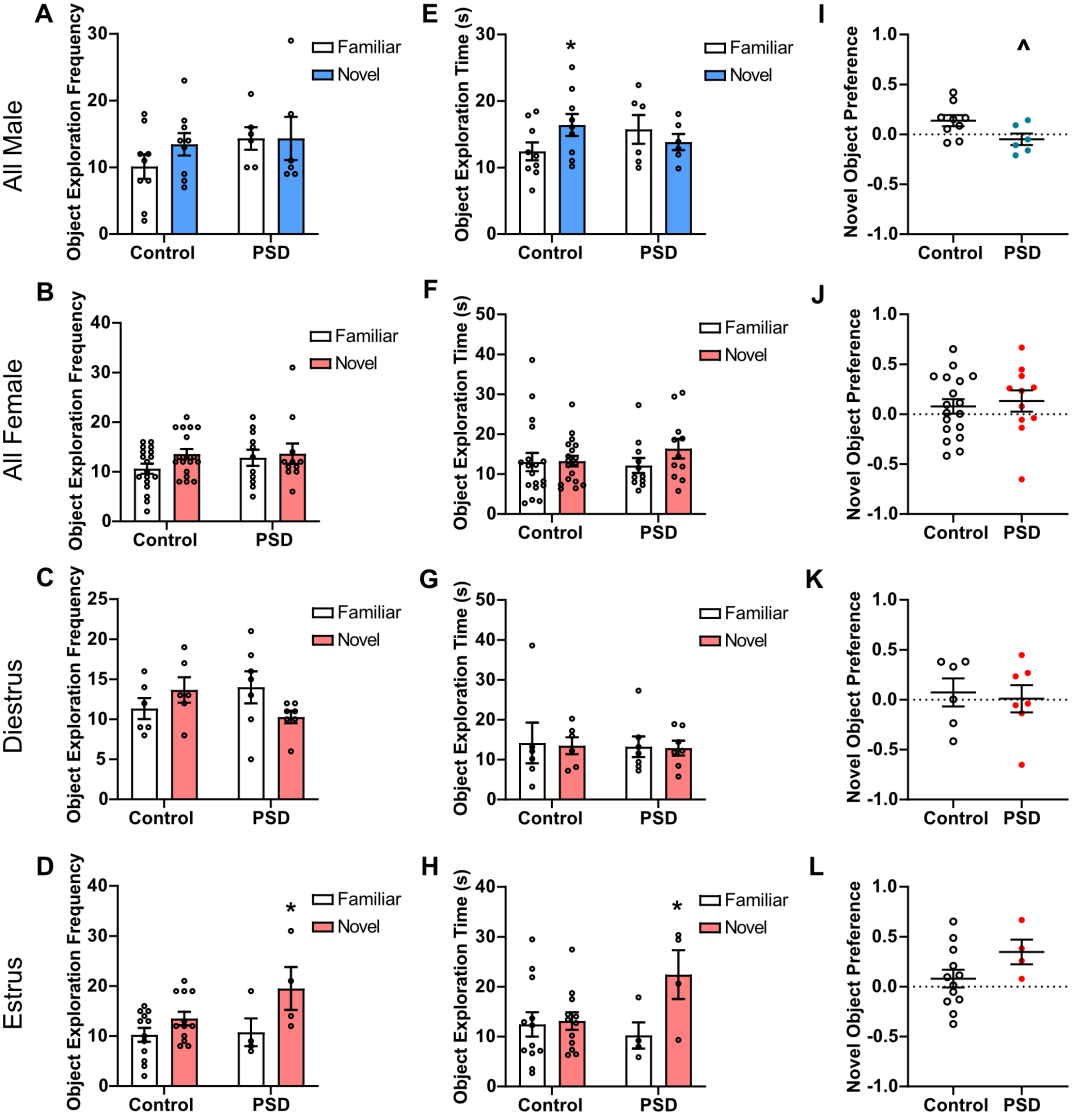
Novel Object Recognition (NOR) performance after paradoxical sleep deprivation (PSD): sex and estrous cycle mediate PSD effects. **(A)** No significant effects of PSD before NOR testing were found for object exploration frequency by a two-way analysis of variance (ANOVA) for all male subjects. **(B)** A two-way ANOVA also found no significant effect for object exploration frequency for all female subjects and **(C)** females in diestrus. **(D)** For females in estrus, a significant main effect of ‘Object' for frequency of object exploration was found by two-way ANOVA. A *post hoc* analysis found that this effect was significant for the PSD estrus females. **(E)** A two-way ANOVA found a significant interaction between group and object for object exploration time in male subjects. Post-hoc analysis found the effect was significant among the control animals. **(F)** No significant effects of PSD were found for object exploration time by two-way ANOVA for all female subjects and **(G)** females in diestrus. **(H)** A two-way ANOVA found a significant interaction between group and object and a significant main effect of ‘Object' for object exploration time for females in the estrus phase. *Post hoc* analysis found the effect was significant among the PSD animals. **(I)** PSD before NOR testing caused a decline in novel object preference (NP) score for male subjects. **(J)** PSD did not affect NP for all female subjects and for females in **(K)** diestrus or **(L)** estrus phase. Bar graph and scatterplot data are presented as mean ± standard error of the mean (SEM) for all males (control *n* = 9, PSD *n* = 6), all females (control *n* = 18, PSD *n* = 11), diestrus females (control *n* = 6, PSD *n* = 7), and estrus females (control *n* = 12, PSD *n* = 4). See Supplementary Tables S3, S4 for full statistical results. *p < 0.05 vs. familiar object. Λ*p* < 0.05 vs. control.

When the familiar and novel objects were introduced on the NOR testing day, there was no significant difference in object exploration frequency for PSD male rats (Figure 3A) or PSD female rats (Figure 3B). These effects were mediated by estrous cycle stage in the female rats, as there was no difference in the frequency of object exploration for PSD females in diestrus, but PSD females in estrus more frequently explored the novel object (Figures 3C, D). For object exploration time, a two-way ANOVA found a statistically significant interaction between the effects of group and object in male rats. *Post hoc* analysis revealed that the male control group spent significantly more time exploring the novel object than the familiar object (Figure 3E). There was also a decreasing trend in novel object exploration time for the male PSD group. There was no significant difference in the time spent with the familiar or novel object among all female subjects (Figure 3F). Similar to the frequency of object exploration, the time exploring objects was also estrous cycle–dependent. There was no significant difference in the time with the novel or familiar object for control and PSD females in diestrus (Figure 3G). In contrast, only PSD females in estrus spent significantly more time with the novel object compared to the familiar object (Figure 3H). When normalized for total exploration time, there was a significant decrease in NP for the male PSD group compared to the controls (Figure 3I). NP was not significantly different among the female rats regardless of estrous cycle (Figures 3J–L).

**Figure 4:**
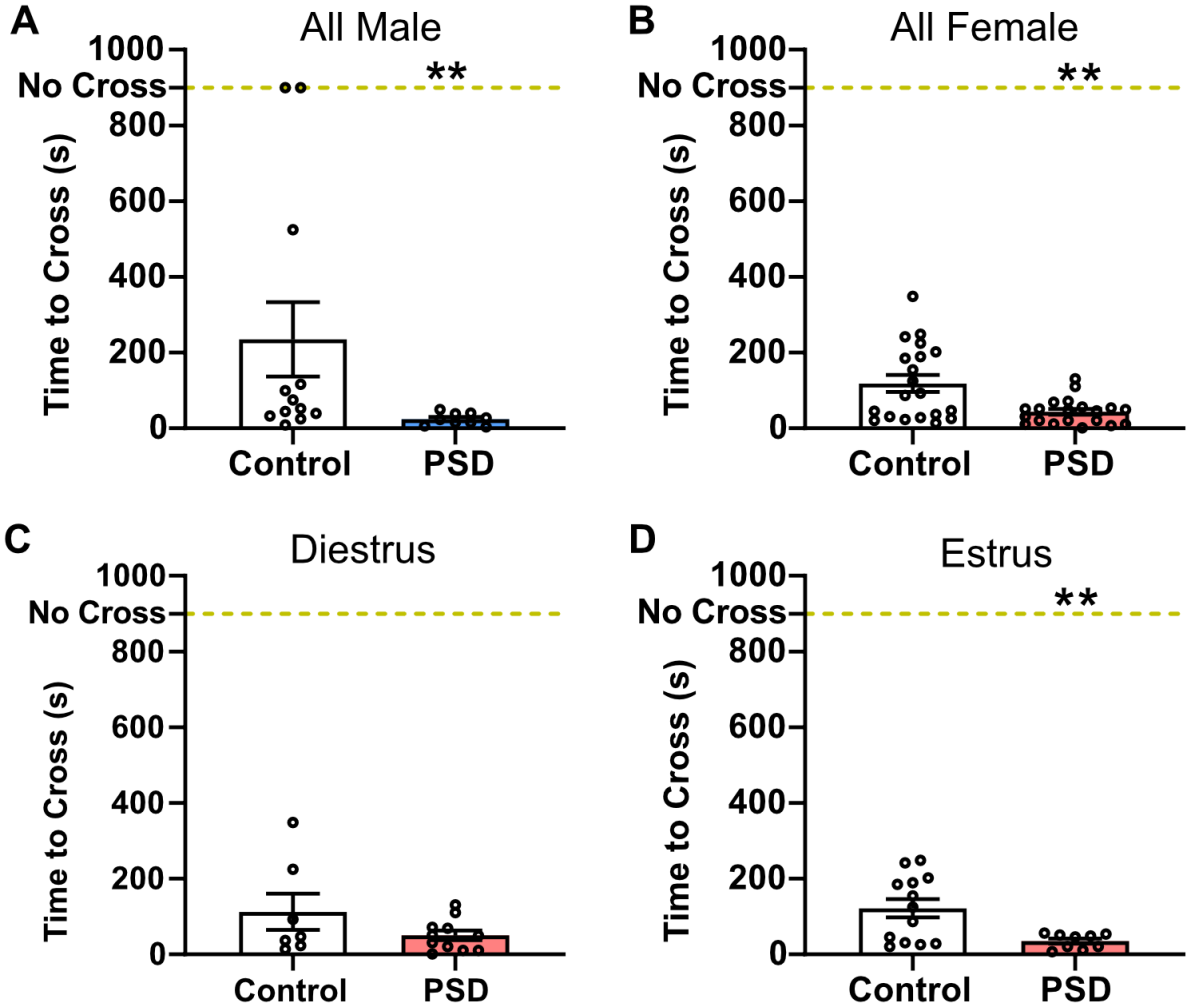
Passive Avoidance Task (PAT) performance after paradoxical sleep deprivation (PSD) is not dependent on sex and influenced by estrous cycle. **(A)** PSD decreased PAT performance in all male subjects. **(B)** PAT performance was also decreased among all female PSD rats. **(C)** No change in PAT performance was found among females in diestrus. **(D)** The decline in PAT performance was maintained in female PSD rats in the estrus phase. Scatterplot data are presented as mean ± standard error of the mean (SEM) for all males (control n = 12, PSD n = 9), all females (control *n* = 20, PSD *n* = 20), diestrus females (control *n* = 7, PSD *n* = 11), and estrus females (control *n* = 13, PSD *n* = 9). See Supplementary Table S5 for full statistical results. **p < 0.01 vs. control.

The PAT was used to examine the effect of PSD on associative aversion learning and memory. For male PSD rats, PAT performance significantly declined when compared to the control group (Figure 4A). Similarly, all PSD females performed worse in the PAT as compared to control female rats (Figure 4B). PAT performance was also influenced by estrous cycle stage because there was no significant difference between the control and PSD females in diestrus (Figure 4C). In contrast, PSD females in estrus were found to have decreased PAT performance compared to the control estrus group (Figure 4D). Although the estrous cycle stage influenced NOR and PAT behavioral outcomes among sleep-deprived females, comparisons between females in diestrus and estrus in the control group indicate that estrous cycle alone does not modulate behavior in these paradigms (Supplementary Figures S2E–I).

### 3.3 Hippocampal-specific changes related to learning and memory performance

**Figure 5:**
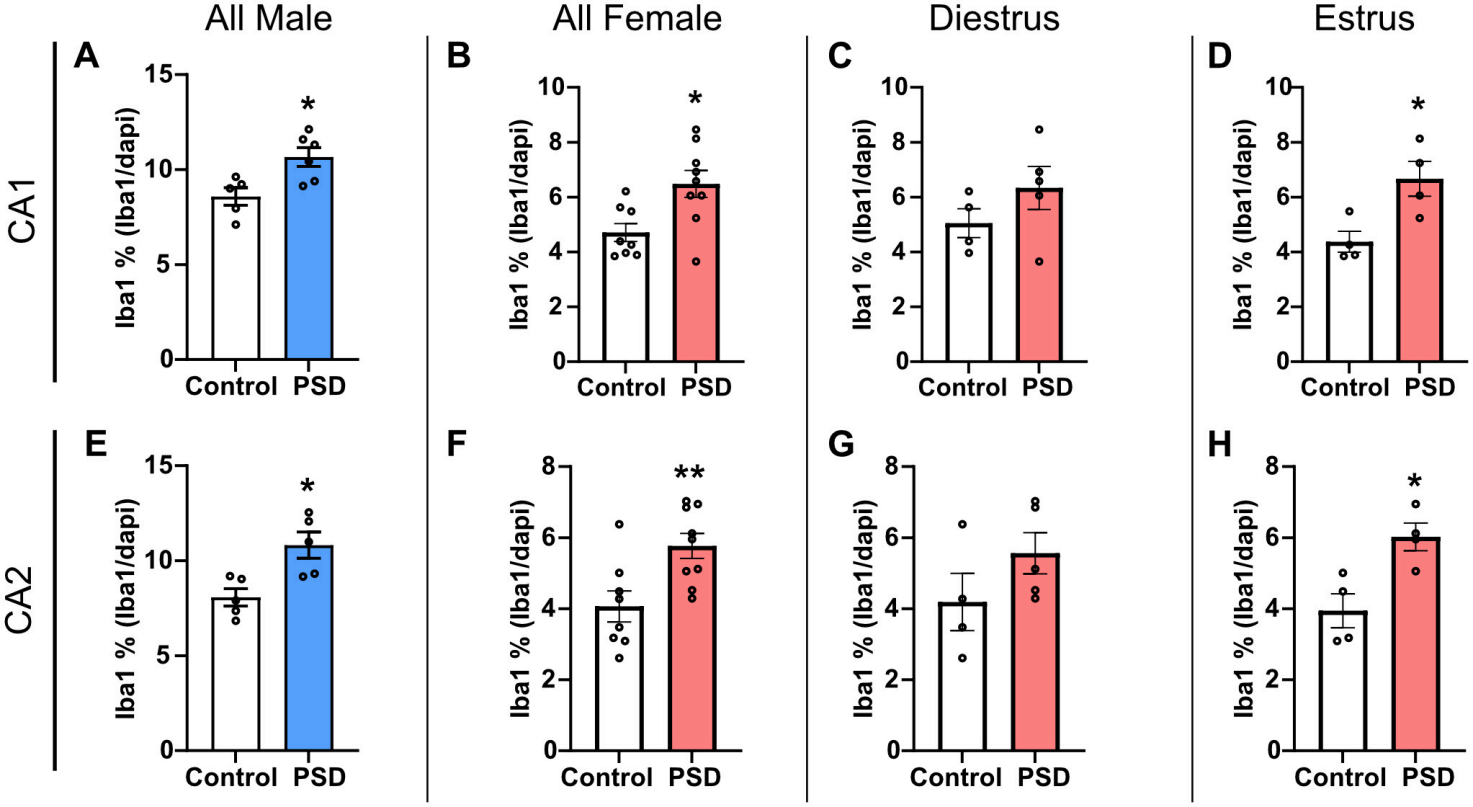
Ionized calcium binding adaptor molecule (Iba1) expression in the hippocampus increases after 120 h of paradoxical sleep deprivation (PSD). **(A)** Iba1 expression increased in the CA1 stratum radiatum (SR) in all males rats after PSD. **(B)** PSD increased Iba1 expression in the CA1 SR in all female rats. **(C)** There was no change in Iba1 expression among female rats in the diestrus phase, while **(D)** the Iba1 increase in the CA1 SR was maintained for female PSD rats in estrus. **(E)** Iba1 expression in the CA2 SR was significantly increased between control and PSD male subjects. **(F)** PSD also induced an increase in CA2 SR Iba1 among all female rats. **(G)** No significant difference in Iba1 expression was found in the CA2 SR for females in diestrus. **(H)** PSD increased Iba1 expression in the CA2 SR among females in the estrus phase. Bar graph data are presented as mean ± SEM for all males (control *n* = 5, CA1 PSD *n* = 6, CA2 PSD *n* = 5), all females (control *n* = 8, PSD *n* = 9), diestrus females (control *n* = 4, PSD *n* = 5), estrus females (control *n* = 4, PSD *n* = 4). See Supplementary Table S8 for full statistical results. *p < 0.05 vs. control. **p < 0.01 vs. control.

To investigate glial cell expression changes associated with decreased learning and memory performance after PSD, IHC was conducted to determine changes in the microglia marker Iba1 and astroglia marker GFAP in the hippocampus. Iba1 expression in the CA1 SR was increased in the PSD groups for both all male and all female subjects when compared to the respective control groups (Figures 5A, B). A Spearman correlation analysis found that as Iba1 expression in the CA1 SR increased, there was a significant increase in time spent within the open arm in the EZM test (*r* = 0.70, *p* = 0.04). However, this correlational relationship between hippocampal Iba1 expression and anxiolytic-like behavior in the EZM was not present among the females (r = 0.18, *p* = 0.51). While there was a trending increase in CA1 SR Iba1 expression among PSD females in diestrus, the increased expression was only significant for PSD females in estrus (Figures 5C, D). PSD also significantly increased Iba1 expression in the CA2 SR when compared to controls for both male and all female subjects (Figures 5E, F). Increased Iba1 expression in the CA2 SR was also estrous cycle–dependent as there was no significant difference between the control and PSD diestrus groups, while the increased Iba1 was maintained for PSD estrus females (Figures 5G, H). An analysis of Iba1 expression in the PL of CA1 and CA2 found no significant changes in expression between the control and PSD groups for either sex or estrous cycle (Supplementary Figures S4–S5). Similarly, PSD did not change the total GFAP expression in the SR or PL in the CA1 or CA2 based on sex or estrous cycle stage (Supplementary Figures S6–S8).

## 4 Discussion

In this study, we aimed to characterize the behavioral effects of PSD in male and female rats with a focus on cognitive outcomes. Overall, disrupted sleep led to anxiolytic-like effects, impaired learning, and hippocampal microgliosis in males and females in estrus. The results suggest that although there are sex-specific differences in paradoxical PSD outcomes, the primary mediator underlying these sex-specific differences is estrous cycle–dependent effects among females. An interaction between estrous cycle stage and paradoxical PSD determines behavioral and neuroinflammatory outcomes among females. Although results suggest that microgliosis in the hippocampus may be a factor in the manifestation of PSD-induced behavioral effects among males and females in estrus, whether this association is causative or symptomatic of other upstream pathways requires further investigation.

Previous animal research on anxiety-like behavior after PSD is laden with inconsistent findings (Vollert et al., 2011; Hajali et al., 2012; Pires et al., 2016). A meta-analysis concluded that preclinical models of PSD decrease anxiety-like behavior (Pires et al., 2016), and the consensus of the analysis aligns with the present study's findings. However, they caution the translatability of traditional anxiety paradigms used in preclinical studies for sleep research purposes (Pires et al., 2016). Intriguingly, a previous study found that exercise modulated the PSD effects on anxiety-like behavior (Vollert et al., 2011). The males in the present study exhibited consistent increased locomotor activity in EZM and NOR, suggesting that the opportunity for exercise based on the PSD paradigm type may explain our results and inconsistencies in previous preclinical studies. However, an increase in anxiolytic-like behavior without increased locomotor activity among females in estrus suggests sex- and estrous-specific differences mediate sleep disruption–induced anxiety-like behavioral outcomes. Previous studies comparing sex-specific differences found females to be more susceptible to increased anxiety-like behavior after PSD (Hajali et al., 2012; Gonzalez-Castañeda et al., 2016). Although a few studies have investigated the estrous cycle's influence on anxiety-like behavior in rodents, some studies have reported reduced anxiety-like behavior among females in proestrus/estrus (Molina-Hernández et al., 2013; Rodríguez-Landa et al., 2021; Marcondes et al., 2001). Although this study did not replicate this finding when comparing diestrus and estrus females in the control group, this previously described phenomenon might explain the interaction found between estrous cycle stage and PSD.

Previous research on the effects of PSD on learning and memory is also inconsistent, especially when sex- and estrous-dependent effects are investigated (Hajali et al., 2012; Fernandes-Santos et al., 2012; Cordeira et al., 2018). The influence of sex on PSD-induced cognitive impairment appears to be task dependent. Previous studies found females to be more susceptible to PSD-induced cognitive performance impairment in the Morris Water Maze and discrimination tasks (Hajali et al., 2012; Fernandes-Santos et al., 2012). However, in alignment with this study's findings, no sex specific difference in PAT performance after PSD was found (Fernandes-Santos et al., 2012). Similar to the literature, the overall cognitive effects of PSD were not consistent among females between NOR and PAT. This could be due, in part, to the lack of preference for the novel object among the control group's females. A change toward impaired performance in NOR behavior cannot be detected if the control group does not demonstrate a novel object preference. This suggests that the NOR paradigm in this study might need to be modified (such as increasing the amount of exploration time during training) for this group. Alternatively, it is possible that the PSD paradigm is not equivalent between male and female rodents. A limitation of this study is that sleep architecture was not tracked. It is possible that the female rodents experienced more microsleeps during PSD than males, possibly leaving them more resilient to measurable cognitive decline from PSD. Although not replicated in this study, a previous study found that females in estrus performed better in a discrimination task compared to females in diestrus abolished by PSD (Cordeira et al., 2018). The present study's finding that estrous cycle mediates the effect of PSD on fear-aggravated learning in PAT has not previously been investigated to our knowledge. Although previous studies implicate estrogen as neuroprotective, these studies were often conducted in ovariectomized females or disease conditions, with conclusions that may not apply to the context of PSD (Morale et al., 2006; Morales et al., 2006; Gould et al., 1990).

Glial cell expression in hippocampal subregions was investigated after paradoxical PSD to determine if there are sex- or estrous cycle–dependent effects and if these effects parallel the neurobehavioral findings. The Iba1 results indicate that PSD-induced microgliosis patterns in the CA1/2 corresponded to multiple neurobehavioral outcomes. The same males and females in estrus that exhibited increased anxiolytic-like behavior and cognitive impairment in a fear-aggravated learning task had increased microglia presence in the CA1/2. A previous study in rats also found hippocampal microgliosis and increased pro-inflammatory cytokines after PSD that was associated with impaired spatial memory performance (Wadhwa et al., 2017).

The novel contribution of the present study is the finding that paradoxical PSD-induced microgliosis is only sex-specific in the context of estrous cycle–dependent effects. Previous studies indicate that microglia have estrogen receptors and that exposure to estrogen hormones modulates microglia expression of immune-regulating major histocompatibility complex-I (MHC-1) (Dimayuga et al., 2005). Based on the literature, estrogen-hormone-mediated suppression of MHC-1 would prevent microglia synaptic pruning (Dimayuga et al., 2005; Huh et al., 2000). Under certain circumstances, although preventing microglia synaptic pruning may be neuroprotective, in the context of PSD and sleep-associated homeostatic synaptic plasticity, a suppression of microglia synaptic pruning may hinder normal memory consolidation processes (Huh et al., 2000; Tononi and Cirelli, 2006, 2014). Further research is required to better understand the association between hippocampal microgliosis and PSD neurobehavioral outcomes, especially in the context of sex- and estrous-specific differences.

Overall, the present study contributes to a better foundational understanding of sex- and estrous-specific outcomes after PSD. The inclusion of female subjects and investigating estrous cycle effects is still relatively in its infancy. In addition to contributing to the field of sleep research, this study further supports the need to reexamine neurobehavioral concepts with a focus on sex-specific and estrous cycle investigation.

## Data Availability

The raw data supporting the conclusions of this article will be made available by the authors, without undue reservation.
