# Supplementary material for: Sex- and estrous-specific effects of paradoxical sleep deprivation: neurobehavioral changes and hippocampal neuroinflammation
Source: Front Sleep. 2025 Sep 22;4:1611192. doi: 10.3389/frsle.2025.1611192 (PMC12713950; doi:10.3389/frsle.2025.1611192)
Supplement: Supplementary file 1 [file Data_Sheet_1.docx]

**Supporting Material for**

Sex- and estrous-specific effects of paradoxical sleep deprivation: Neurobehavioral changes and hippocampal neuroinflammation

Laura K. Olsen^1,2^, Krysten A. Jones^1,3^, Raquel J. Moore^1,4^, Hunter McCubbins^1,2^, Frances S. Curtner^1,4^, Birendra Sharma^1,2^, and Candice N. Hatcher-Solis^1^

* Correspondence should be addressed to:

laura.olsen.1.ctr@us.af.mil

**Table of Contents**

Figure S1 S2

Figure S2 S3

Figure S3-S4 S4

Figure S5 S5

Figure S6 S6

Figure S7 S7

Figure S8 S8

Table S1 S9

Table S2 S10

Table S3 S10-11

Table S4 S12

Table S5 S12

Table S6 S13

Table S7 S13

Table S8 S14

Table S9 S15


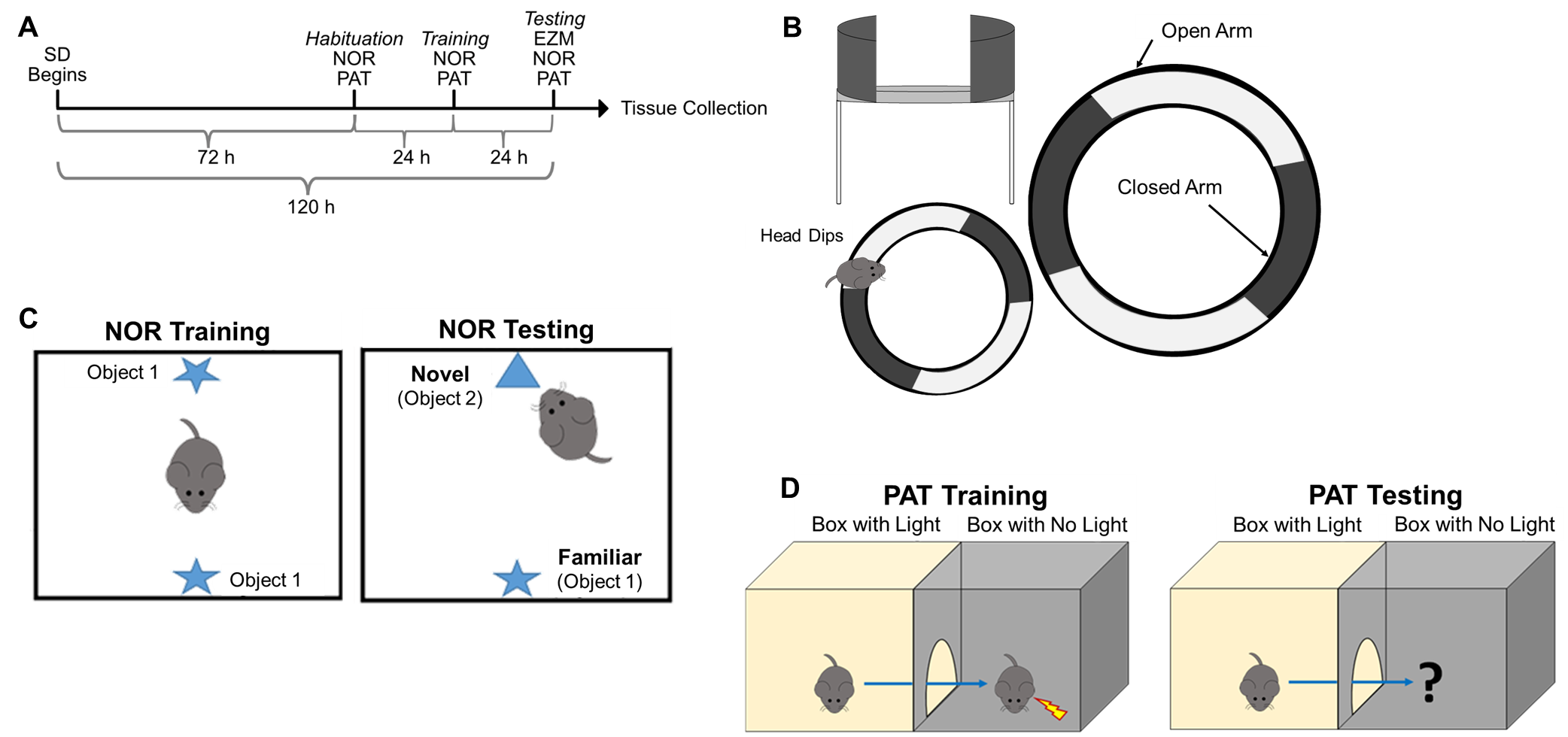


**Figure S1. Overview of experimental design.** (A) Male and female Sprague-Dawley rats underwent a total of 120 h of sleep deprivation (PSD) during behavioral testing. After 72 h of PSD, animals were habituated to the NOR and PAT arenas on habituation day. The following day, after an additional 24 h of PSD, training for the NOR and PAT tasks was completed. Testing for the (B) EZM, (C) NOR, and (D) PAT paradigms was performed after an additional 24 h of PSD. Animals were euthanized and brain tissue was collected after PSD and behavioral testing for immunohistochemistry (IHC). Control animals were subjected to the same experimental timeline but control platforms were utilized that do not restrict sleep.

**
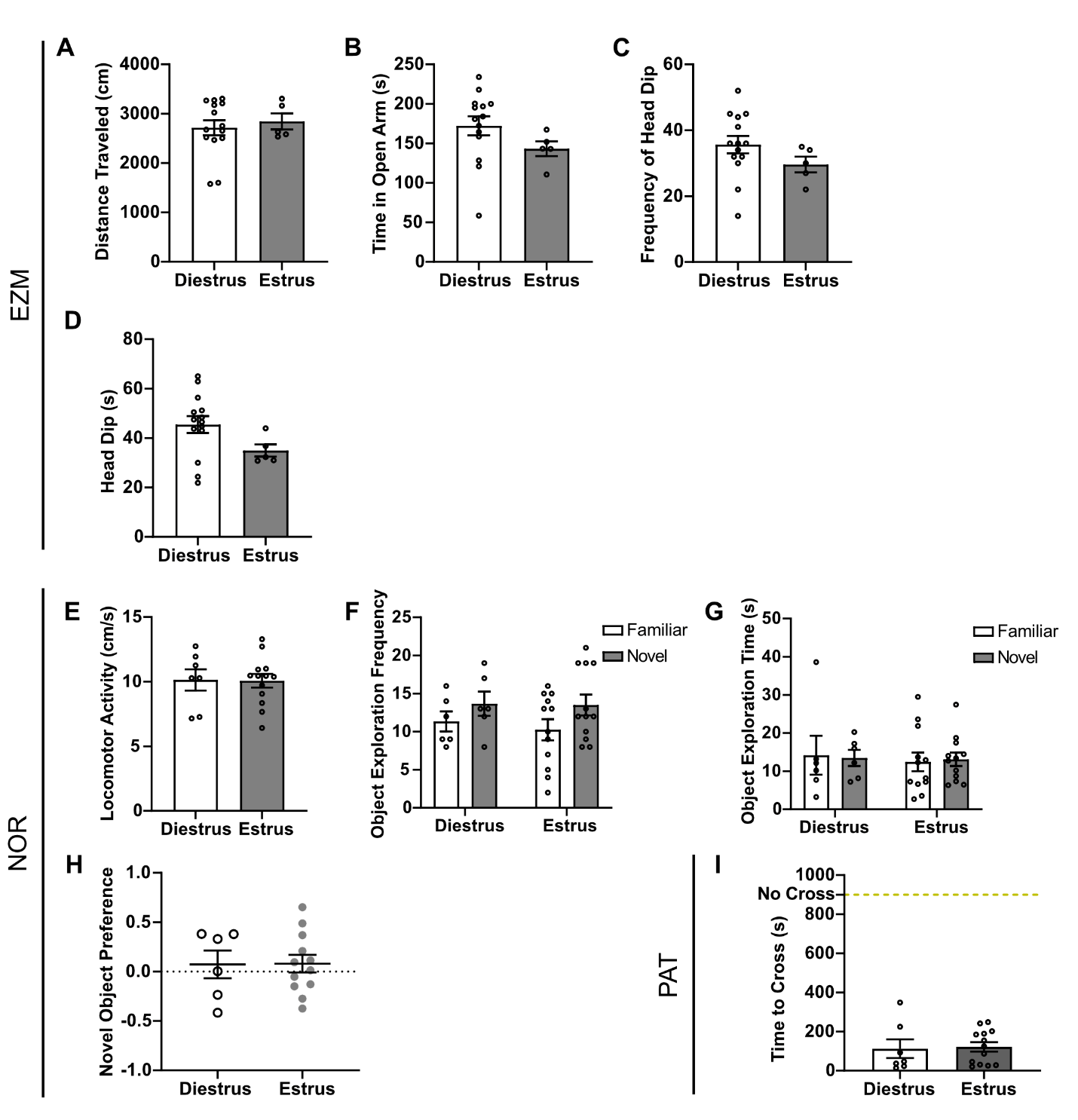
**

**Figure S2. Behavior outcomes are not significantly different between estrous cycle stage for control animals.** (A-D) Measurements of anxiety-like behavior as assessed by EZM did not change between control diestrus (n = 14) and control estrus (n = 5) females. (F-I) Estrous cycle did not change locomotor activity (n = 7 diestrus, n = 13 diestrus) or NOR performance (n = 6 diestrus, n = 12 estrus) among female controls. (J) PAT performance as not statistically different between diestrus controls (n = 7) and estrus (n = 13) controls. Bar graph and scatter plot data are presented as mean ± SEM.


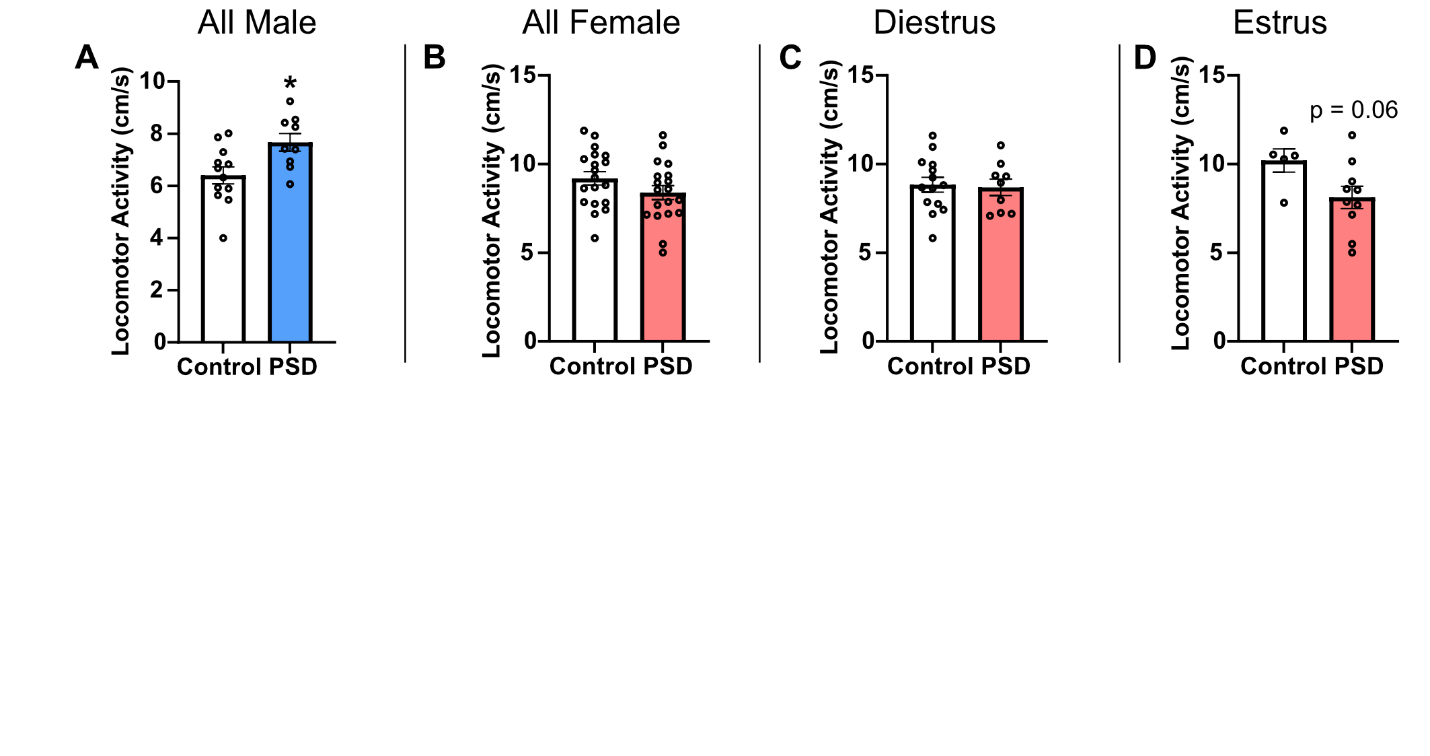


**Figure S3. Locomotor activity on NOR habituation day.** (A) Locomotor activity was increased for male PSD rats compared to controls. (B) PSD did not affect locomotor activity in all female subjects or for (C) females in diestrus. (D) A trending decrease in locomotor activity was found for females in estrus. Bar graph data are presented as mean ± SEM for all males (control n = 12, PSD n = 9), all females (control n = 19, PSD n = 19), diestrus females (control n = 14, PSD n = 9), and estrus females (control n = 5, PSD n = 10). *p < 0.05 vs control.

**
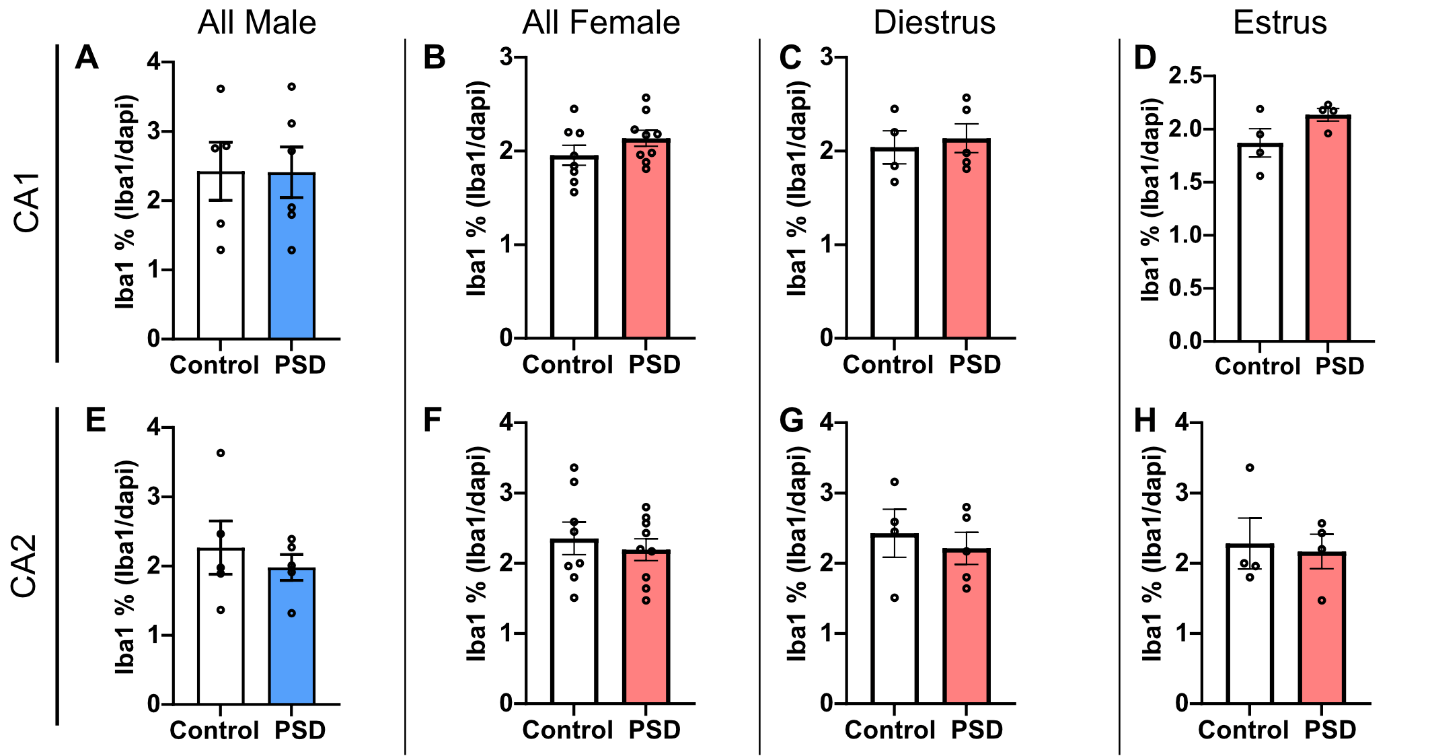
**

**Figure S4. Hippocampal pyramidal layer (PL) Iba1 expression is not affected by 120 h PSD.** (A) There was no change in Iba1 expression in the hippocampal CA1 PL between male control and PSD rats. (B-D) PSD did not induce Iba1 expression among all females or based on estrous cycle stage. (E) No significant difference in Iba1 expression was found in the CA2 PL for male rats. (F-H) There was no difference in Iba1 expression between control and PSD female rats regardless of estrous cycle stage. Bar graph data are presented as mean ± SEM for all males (control n = 5, CA1 PSD n = 6, CA2 PSD n = 5), all females (control n = 8, PSD n = 9), diestrus females (control n = 4, PSD n = 5), estrus females (control n = 4, PSD n = 4).


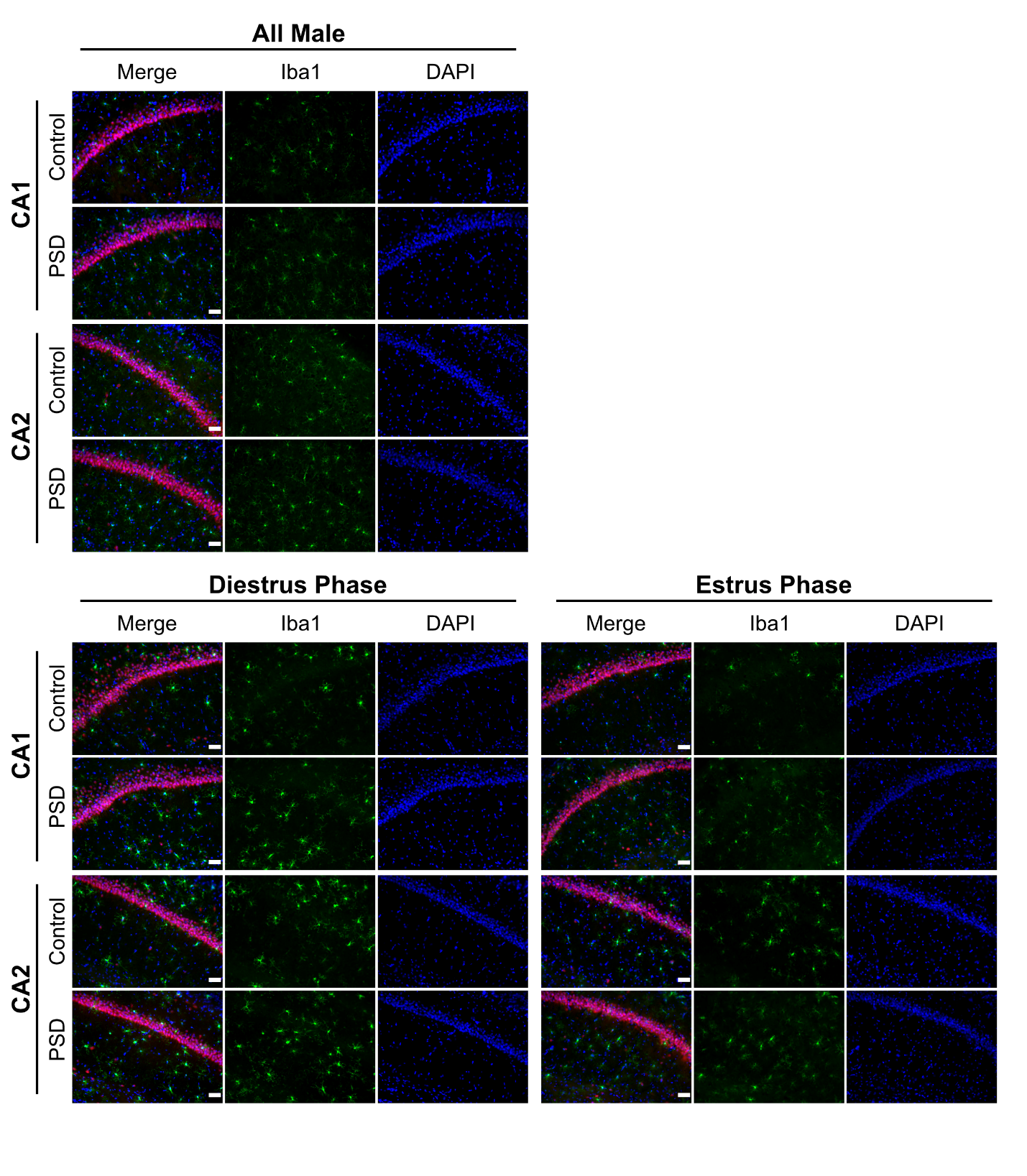


**Figure S5. Hippocampal Iba1 expression 120 h after PSD.** Representative IHC images of Iba1 expression in the CA1 and CA2 as quantified in Figure 5 and Supplemental Figure 3. Merge images show Iba1 (green), NeuN (red), and DAPI (blue) staining. Scale bar = 50 µm.

**
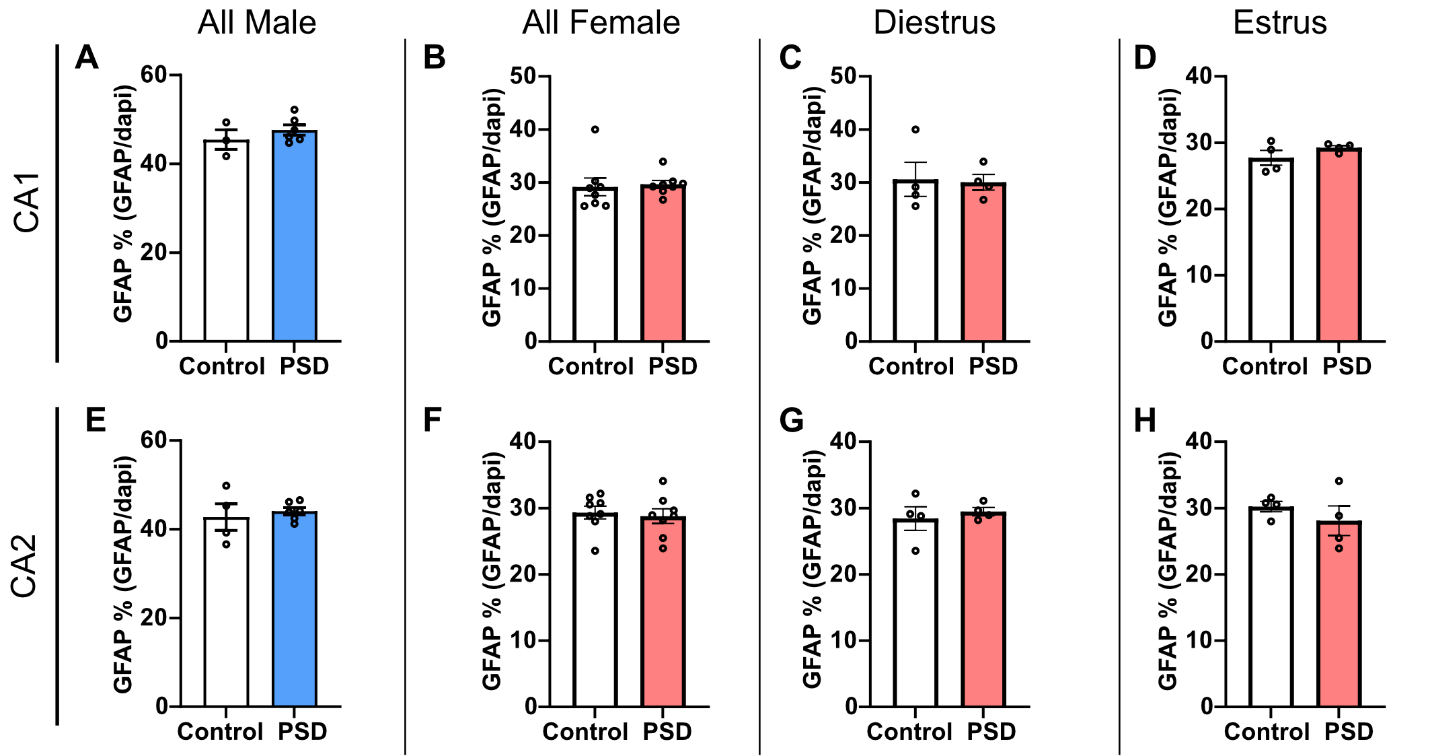
**

**Figure S6. GFAP expression in hippocampal SR is not affected by 120 h PSD.** (A) There was no change in GFAP expression in the SR of the CA1 between male control and PSD rats. (B-D) PSD did not affect GFAP expression in the CA1 SR among all females or based on estrous cycle stage. (E) No significant difference in GFAP expression was found in the CA2 PL for male rats. (F-H) There was no difference in GFAP expression between control and PSD female rats regardless of estrous cycle stage. Bar graph data are presented as mean ± SEM for all males (CA1 control n = 3, CA2 control n = 4, PSD n = 6), all females (control n = 8, PSD n = 8), diestrus females (control n = 4, PSD n = 4), estrus females (control n = 4, PSD n = 4).

**
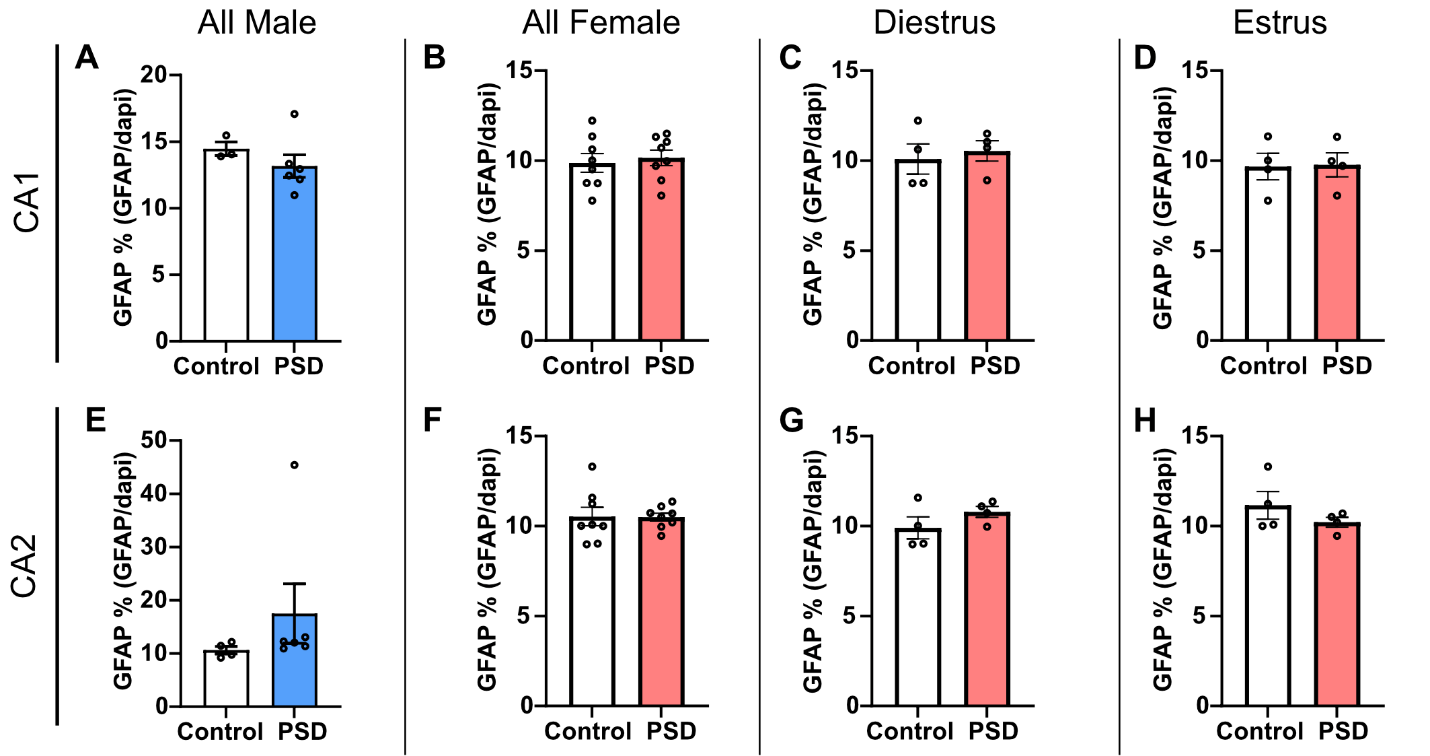
**

**Figure S7. Hippocampal PL GFAP expression is not affected by 120 h PSD.** (A-B) PSD did not change the total GFAP expression in the PL of the CA1 in male rats or female rats. (C-D) When estrous cycle was considered, there was no change in GFAP expression between control or PSD female rats in the CA1 PL. (E) There was no significant difference in GFAP expression in the CA2 PL between control and PSD male rats. (F-H) PSD did not affect GFAP expression in the CA2 PL among all females or based on estrous cycle stage. Bar graph data are presented as mean ± SEM for all males (CA1 control n = 3, CA2 control n = 4, PSD n = 6), all females (control n = 8, PSD n = 8), diestrus females (control n = 4, PSD n = 4), estrus females (control n = 4, PSD n = 4).


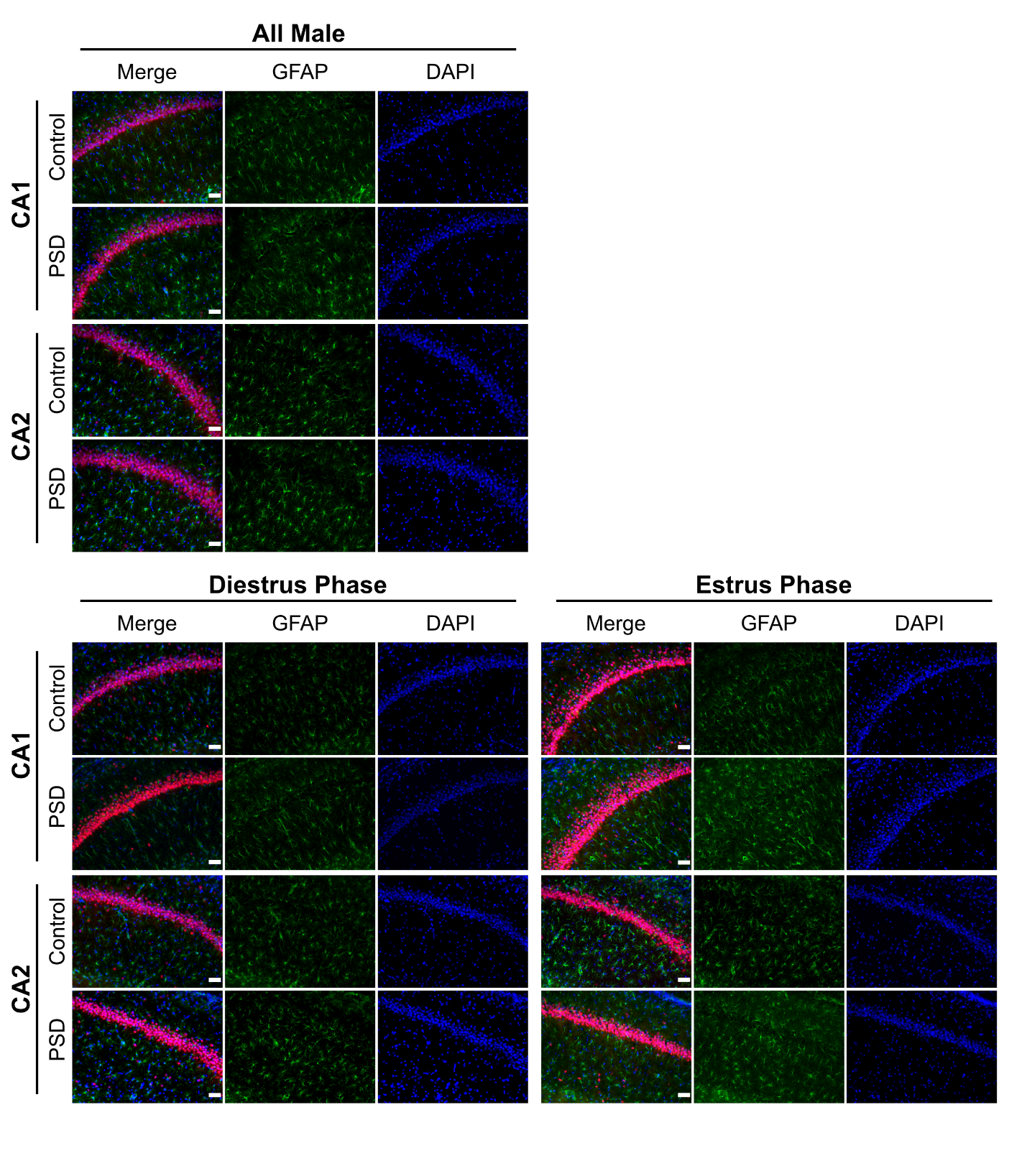


**Figure S8. Hippocampal GFAP expression 120 h after PSD.** Representative IHC images of GFAP expression in the CA1 and CA2 as quantified in Supplemental Figure 5-6. Merge images show GFAP (green), NeuN (red), and DAPI (blue) staining. Scale bar = 50 µm.

| **Analysis** | **T** | **df** | **p-value** | **95% CI of difference** | **Mean±SEM** |
| --- | --- | --- | --- | --- | --- |
| ***Elevated Zero Maze- Male, All Subjects*** | | | | | |
| Distance Traveled | 4.629 | 19 | < 0.001 | 382.8 – 1015 | Control = 1781±90.74  PSD = 2480±125.8 |
| Time in Open Arm(s) | 2.967 | 19 | 0.008 | 14.16 – 81.96 | Control = 121.6±11.82  PSD = 169.7±9.987 |
| Head Dip Frequency | 3.957 | 19 | < 0.001 | 2.931 – 9.513 | Control = 11.67±1.170  PSD = 17.89±0.9196 |
| Head Dip Time | 4.358 | 19 | < 0.001 | 6.011 – 17.12 | Control = 17.60±1.930  PSD = 29.17±1.652 |
| ***Elevated Zero Maze- Female, All Subjects*** | | | | | |
| Distance Traveled | 1.053 | 34 | 0.300 | -169.0 – 532.5 | Control = 2749±118.4  PSD = 2930±125.7 |
| Time in Open Arm(s) | 1.971 | 34 | 0.057 | -0.7369 – 47.80 | Control = 164.6±9.577  PSD = 188.2±6.677 |
| Head Dip Frequency | 0.754 | 34 | 0.456 | -3.698 – 8.063 | Control = 34.05±2.098  PSD = 36.24±1.964 |
| Head Dip Time | 1.107 | 34 | 0.276 | -4.014 – 13.63 | Control = 42.66±2.787  PSD = 47.47±3.372 |
| ***Elevated Zero Maze- Diestrus Phase*** | | | | | |
| Distance Traveled | 0.886 | 25 | 0.384 | -254.8 – 639.6 | Control = 2715±152.1  PSD = 2907±154.8 |
| Time in Open Arm(s) | 0.851 | 25 | 0.403 | -17.64 – 42.49 | Control = 172.3±12.07  PSD = 184.7±7.758 |
| Head Dip Frequency | 0.123 | 25 | 0.903 | -6.823 – 7.691 | Control = 35.64±2.628  PSD = 36.08±2.313 |
| Head Dip Time | 0.296 | 25 | 0.770 | -9.694 – 12.95 | Control = 45.42±3.418  PSD = 47.05±4.360 |
| ***Elevated Zero Maze- Estrus Phase*** | | | | | |
| Distance Traveled | 0.623 | 7 | 0.553 | -453.1 – 777.5 | Control = 2843±161.6  PSD = 3005±210.3 |
| Time in Open Arm(s) | 3.593 | 7 | 0.0088 | 19.21 – 93.14 | Control = 143.2±9.274  PSD = 199.4±13.17 |
| Head Dip Frequency | 1.562 | 7 | 0.162 | -3.674 – 17.97 | Control = 29.6±2.379  PSD = 36.75±4.211 |
| Head Dip Time | 3.527 | 7 | 0.0096 | 4.583 – 23.22 | Control = 34.94±2.489  PSD = 48.84±3.133 |

**Table S1.** Statistical analyses for EZM behavioral data.

| **Analysis** | **t** | **df** | **p-value** | **95% CI of difference** | **Mean±SEM** |
| --- | --- | --- | --- | --- | --- |
| ***Novel Object Recognition- Locomotor Activity (Testing Day)*** | | | | | |
| Male, All Subjects | 3.113 | 19 | 0.006 | 1.041 – 5.314 | Control = 5.041±0.5848  PSD = 8.218±0.8873 |
| Female, All Subjects | 0.749 | 38 | 0.458 | -1.821 – 0.8372 | Control = 10.09±0.4331  PSD = 9.602±0.4932 |
| Diestrus Phase | 0.651 | 16 | 0.525 | -2.798 – 1.484 | Control = 10.14±0.8228  PSD = 9.479±0.6135 |
| Estrus Phase | 0.340 | 20 | 0.737 | -2.275 – 1.637 | Control = 10.07±0.5240  PSD = 9.751±0.8390 |
| ***Novel Object Recognition- Locomotor Activity (Testing Day)*** | | | | | |
| Male, All Subjects | 2.665 | 19 | 0.015 | 0.271 – 2.259 | Control = 6.402  PSD = 7.668 |
| Female, All Subjects | 1.494 | 36 | 0.144 | -1.905 – 0.289 | Control = 9.195  PSD = 8.387 |
| Diestrus Phase | 0.519 | 19 | 0.611 | -1.754 – 1.059 | Control = 9.034  PSD = 8.687 |
| Estrus Phase | 2.064 | 13 | 0.060 | -4.265 – 0.097 | Control = 10.200  PSD = 8.116 |

**Table S2.** Statistical analyses for NOR behavioral data.

| **Analysis** | **F** | **df** | **p-value** | **95% CI of difference** | **Means±SEM** |
| --- | --- | --- | --- | --- | --- |
| ***Novel Object Recognition- Frequency of Object Exploration, Male All Subjects*** | | | | | |
| Main Effect Object | 0.570 | 1,13 | 0.464 | -6.435 – 3.102 | Familiar = 12.22±2.111  Novel = 13.89±0.444 |
| Main Effect Group | 1.568 | 1,13 | 0.233 | -6.964 – 1.853 | Control = 11.778±1.667  PSD = 14.333±0.00 |
| Interaction: Object and Group | 0.570 | 1,13 | 0.464 | -12.87 – 6.203 | Familiar control = 10.111±1.837 Familiar PSD = 14.333±1.687  Novel control = 13.444±1.676  Novel PSD = 14.333±3.242 |
| ***Novel Object Recognition- Frequency of Object Exploration, Female All Subjects*** | | | | | |
| Main Effect Object | 1.879 | 1,27 | 0.181 | -4.697 – 0.9344 | Familiar = 11.71±1.104  Novel = 13.60±0.0404 |
| Main Effect Group | 0.701 | 1,27 | 0.410 | -3.948 – 1.660 | Control = 12.083±1.472  PSD = 13.227±0.409 |
| Interaction: Object and Group | 0.600 | 1,27 | 0.445 | -7.758 – 3.505 | Familiar Control = 10.611±1.007  Familiar PSD = 12.818±1.617  Novel Control = 13.556±1.027  Novel PSD = 13.636±2.051 |
| ***Novel Object Recognition- Frequency of Object Exploration, Diestrus Phase*** | | | | | |
| Main Effect Object | 0.174 | 1,11 | 0.684 | -2.949 – 4.330 | Familiar = 12.67±1.333  Novel = 11.98±1.690 |
| Main Effect Group | 0.072 | 1,11 | 0.793 | -2.570 – 3.284 | Control = 12.50±1.167  PSD = 12.143±1.857 |
| Interaction: Object and Group | 3.343 | 1,11 | 0.095 | -13.33 – 1.232 | Familiar Control = 11.333±1.308  Familiar PSD = 14.0±2.0  Novel Control = 13.667±1.585  Novel PSD = 10.286±0.778 |
| **Analysis** | **F** | **df** | **p-value** | **95% CI of difference** | **Means±SEM** |
| ***Novel Object Recognition- Frequency of Object Exploration, Estrus Phase*** | | | | | |
| Main Effect Object | 10.06 | 1,14 | 0.0068 | -10.06 – -1.943 | Familiar = 10.50±0.250  Novel = 16.50±3.0 |
| Main Effect Group | 1.724 | 1,14 | 0.210 | -8.558 – 2.058 | Control = 11.875±1.625  PSD = 15.125±4.375 |
| Interaction: Object and Group | 2.114 | 1,14 | 0.168 | -2.613 – 13.61 | Familiar Control = 10.250±1.388  Familiar PSD = 10.750±2.780  Novel Control = 13.50±1.368  Novel PSD = 19.50±4.291 |
| **Analysis** | **F** | **df** | **p-value** | **95% CI of difference** | **Means±SEM** |
| ***Novel Object Recognition- Object Exploration Time, Male All Subjects*** | | | | | |
| Main Effect Object | 0.767 | 1,13 | 0.397 | -3.567 – 1.509 | Familiar = 14.08±1.649  Novel = 15.11±1.287 |
| Main Effect Group | 0.032 | 1,13 | 0.861 | -4.740 – 4.015 | Control = 14.418±1.982  PSD = 14.780±0.953 |
| Interaction: Object and Group | 6.243 | 1,13 | 0.027 | -10.95 – -0.7947 | Familiar Control = 12.436±1.347  Familiar PSD = 15.733±2.161  Novel Control = 16.400±1.660  Novel PSD = 13.827±1.214 |
| ***Novel Object Recognition- Object Exploration Time, Female All Subjects*** | | | | | |
| Main Effect Object | 1.335 | 1,27 | 0.258 | -6.143 – 1.717 | Familiar = 12.58±0.4391  Novel = 14.79±1.559 |
| Main Effect Group | 0.254 | 1,27 | 0.618 | -5.677 – 3.438 | Control = 13.128±0.108  PSD = 14.247±2.105 |
| Interaction: Object and Group | 1.088 | 1,27 | 0.306 | -3.864 – 11.86 | Familiar Control = 13.020±2.271  Familiar PSD = 12.142±1.875  Novel Control = 13.236±1.335  Novel PSD = 16.353±2.452 |
| ***Novel Object Recognition- Object Exploration Time, Diestrus Phase*** | | | | | |
| Main Effect Object | 0.027 | 1,11 | 0.873 | -6.495 – 7.537 | Familiar = 13.71±0.4729  Novel = 13.19±0.3005 |
| Main Effect Group | 0.069 | 1,11 | 0.798 | -5.701 – 7.248 | Control = 13.833±0.347  PSD = 13.060±0.174 |
| Interaction: Object and Group | .0029 | 1,11 | 0.958 | -13.69 – 14.38 | Familiar Control = 14.180±5.095  Familiar PSD = 13.234±2.583  Novel Control = 13.487±2.120  Novel PSD = 12.886±1.848 |
| ***Novel Object Recognition- Object Exploration Time, Estrus Phase*** | | | | | |
| Main Effect Object | 8.142 | 1,14 | 0.013 | -11.26 – -1.597 | Familiar = 11.34±1.105  Novel = 17.77±4.655 |
| Main Effect Group | 0.928 | 1,14 | 0.352 | -11.45 – 4.354 | Control = 12.775±0.335  PSD = 16.325±6.095 |
| Interaction: Object and Group | 6.534 | 1,14 | 0.023 | 1.854 – 21.19 | Familiar Control = 12.440±2.442  Familiar PSD = 10.230±2.640  Novel Control = 13.110±1.762  Novel PSD = 22.420±4.888 |

**Table S3.** Statistical analyses for NOR behavioral data.

| **Analysis** | **t** | **df** | **p-value** | **95% CI of difference** | **Mean±SEM** |
| --- | --- | --- | --- | --- | --- |
| ***Novel Object Preference- Novel Object Preference*** | | | | | |
| Male, All Subjects | 2.234 | 13 | 0.044 | -0.366 – -0.006 | Control = 0.1369±0.0561  PSD = -0.0491±0.0572 |
| Female, All Subjects | 0.435 | 27 | 0.667 | -0.202 – 0.311 | Control = 0.07765±0.07319  PSD = 0.1320±0.1064 |
| Diestrus Phase | 0.327 | 11 | 0.750 | -0.496 – 0.367 | Control = 0.07348±0.1407  PSD = 0.009358±0.1356 |
| Estrus Phase | 1.561 | 14 | 0.141 | -0.0999 – 0.634 | Control = 0.07973±0.08899  PSD = 0.3467±0.1236 |

**Table S4.** Statistical analyses for NOR NP behavioral data.

| **Analysis** | **U** | **p-value** | | **Median latency** | | **Mean±SEM** |
| --- | --- | --- | --- | --- | --- | --- |
| ***Passive Avoidance Task- Time to Cross*** | | | | | | |
| Male, All Subjects | 17 | 0.007 | | Control = 63.70, n=12  PSD = 23.40, n=9 | | Control = 234.9±98.09  PSD = 24.47±5.293 |
| **Analysis** | **T** | **df** | **p-value** | | **95% CI of difference** | **Mean±SEM** |
| ***Passive Avoidance Task- Time to Cross*** | | | | | | |
| Female, All Subjects | 3.189 | 38 | 0.003 | | -122.2 – -27.29 | Control = 118.5±22.18  PSD = 43.70±7.591 |
| Diestrus Phase | 1.513 | 16 | 0.150 | | -148.2 – 24.76 | Control = 112.4±47.90  PSD = 50.64±12.65 |
| Estrus Phase | 2.920 | 20 | 0.009 | | -148.3 – -24.72 | Control = 121.7±24.05  PSD = 35.22±6.583 |

**Table S5.** Statistical analyses for PAT behavioral data.

| **Analysis** | **t** | | **df** | **p-value** | | **95% CI of difference** | **Mean±SEM** |
| --- | --- | --- | --- | --- | --- | --- | --- |
| ***Elevated Zero Maze- Diestrus vs Estrus phase controls*** | | | | | | | |
| Distance Traveled | 0.466 | | 17 | 0.647 | | -451.8 – 708.0 | Diestrus = 2715±152.1  Estrus = 2843±161.6 |
| Time in Open Arm(s) | 1.370 | | 17 | 0.188 | | -73.91 – 15.70 | Diestrus = 172.3±12.07  Estrus = 143.2±9.274 |
| Head Dip Frequency | 1.292 | | 17 | 0.214 | | -15.91 – 3.826 | Diestrus = 35.64±2.628  Estrus = 29.60±2.379 |
| Head Dip Time | 1.749 | | 17 | 0.098 | | -23.13 – 2.160 | Diestrus = 45.42±3.418  Estrus = 34.94±2.489 |
| ***Novel Object Recognition- Diestrus vs Estrus phase controls*** | | | | | | | |
| Locomotor Activity | 0.071 | | 18 | 0.945 | | -2.026 – 1.894 | Diestrus = 10.14±0.8228  Estrus = 10.07±0.5240 |
| Novel Object Preference | 0.039 | | 16 | 0.969 | | -0.333 – 0.3455 | Diestrus = 0.07348±0.1407  Estrus = 0.07973±0.08899 |
| ***Passive Avoidance Task- Diestrus vs Estrus phase controls*** | | | | | | | |
| Time to Cross | 0.196 | | 18 | 0.847 | | -90.92 – 109.6 | Diestrus = 112.4±47.90  Estrus = 121.7±24.05 |
| **Analysis** | **U** | **p-value** | | | **Median latency** | | **Mean±SEM** |
| ***Elevated Zero Maze- Diestrus vs Estrus phase controls*** | | | | | | | |
| Latency to Open Arm | 26 | 0.430 | | | Diestrus = 1.260, n=14  Estrus = 2.240, n=5 | | Diestrus = 1.713±0.4688  Estrus = 2.568±0.9341 |

**Table S6.** Statistical analyses for diestrus vs estrus phase control behavioral data.

| **Analysis** | **F** | **df** | **p-value** | **95% CI of difference** | **Means±SEM** |
| --- | --- | --- | --- | --- | --- |
| ***Novel Object Recognition- Frequency of Object Exploration, Diestrus vs Estrus controls*** | | | | | |
| Main Effect Object | 3.139 | 1,16 | 0.096 | -6.132 – 0.5486 | Familiar = 10.79±0.5417  Novel = 13.58±0.0833 |
| Main Effect Group | 0.161 | 1,16 | 0.693 | -2.675 – 3.925 | Control = 12.50±1.167  PSD = 11.875±1.625 |
| Interaction: Object and Group | 0.084 | 1,16 | 0.775 | -5.764 – 7.597 | Familiar Control = 11.333±1.308  Familiar PSD = 10.250±1.388  Novel Control = 13.667±1.585  Novel PSD = 13.50±1.368 |
| ***Novel Object Recognition- Object Exploration Time, Diestrus vs Estrus controls*** | | | | | |
| Main Effect Object | 2.58x 10^-5^ | 1,16 | 0.996 | -4.856 – 4.880 | Familiar = 13.31±0.870  Novel = 13.30±0.1883 |
| Main Effect Group | 0.10 | 1,16 | 0.756 | -6.041 – 8.158 | Control = 13.833±0.347  PSD = 12.775±0.335 |
| Interaction: Object and Group | 0.088 | 1,16 | 0.770 | -8.373 – 11.10 | Familiar Control = 14.180±5.095  Familiar PSD = 12.440±2.442  Novel Control = 13.487±2.120  Novel PSD = 13.110±1.762 |

**Table S7.** Statistical analyses for diestrus vs estrus phase control NOR data.

| **Analysis** | **t** | **df** | **p-value** | **95% CI of difference** | **Mean±SEM** |
| --- | --- | --- | --- | --- | --- |
| ***Iba1 Expression- CA1, Stratum Radiatum*** | | | | | |
| Male, All Subjects | 3.014 | 9 | 0.015 | 0.5187 – 3.639 | Control = 8.586±0.4620  PSD = 10.66±0.4969 |
| Female, All Subjects | 2.928 | 15 | 0.010 | 0.4820 – 3.062 | Control = 4.710±0.3261  PSD = 6.482±0.4905 |
| Diestrus Phase | 1.287 | 7 | 0.239 | -1.075 – 3.643 | Control = 5.050±0.5231  PSD = 6.334±0.7811 |
| Estrus Phase | 3.094 | 6 | 0.021 | 0.4803 – 4.115 | Control = 4.370±0.3813  PSD = 6.668±0.6373 |
| ***Iba1 Expression- CA2, Stratum Radiatum*** | | | | | |
| Male, All Subjects | 3.319 | 8 | 0.011 | 0.8396 – 4.661 | Control = 8.068±0.4594  PSD = 10.82±0.6894 |
| Female, All Subjects | 3.059 | 15 | 0.008 | 0.5161 – 2.887 | Control = 4.065±0.4365  PSD = 5.767±0.3532 |
| Diestrus Phase | 1.423 | 7 | 0.198 | -0.9099 – 3.659 | Control = 4.188±0.8065  PSD = 5.562±0.5794 |
| Estrus Phase | 3.379 | 6 | 0.015 | 0.5737 – 3.586 | Control = 3.943±0.4785  PSD = 6.023±0.3873 |
| ***Iba1 Expression- CA1, Pyramidal Layer*** | | | | | |
| Male, All Subjects | 0.022 | 9 | 0.983 | -1.265 – 1.241 | Control = 2.423±0.4196  PSD = 2.411±0.3657 |
| Female, All Subjects | 1.335 | 15 | 0.202 | -0.108 – 0.4687 | Control = 1.955±0.1070  PSD = 2.136±0.085 |
| Diestrus Phase | 0.411 | 7 | 0.693 | -0.456 – 0.6479 | Control = 2.040±0.1757  PSD = 2.136±0.1544 |
| Estrus Phase |  |  |  |  |  |
| ***Iba1 Expression- CA2, Pyramidal Layer*** | | | | | |
| Male, All Subjects | 0.673 | 8 | 0.520 | -1.270 – 0.6960 | Control = 2.266±0.3832  PSD = 1.979±0.1865 |
| Female, All Subjects | 0.589 | 15 | 0.565 | -0.747 – 0.4235 | Control = 2.354±0.2325  PSD = 2.192±0.1563 |
| Diestrus Phase | 0.544 | 7 | 0.603 | -1.152 – 0.7213 | Control = 2.428±0.3422  PSD = 2.212±0.2276 |
| Estrus Phase | 0.257 | 6 | 0.806 | -1.183 – 0.9578 | Control = 2.280±0.3626  PSD = 2.168±0.2447 |

**Table S8.** Statistical analyses for IHC Iba1 data.

| **Analysis** | **t** | | **df** | **p-value** | | **95% CI of difference** | | **Mean±SEM** |
| --- | --- | --- | --- | --- | --- | --- | --- | --- |
| ***GFAP Expression- CA1, Stratum Radiatum*** | | | | | | | | |
| Male, All Subjects | 0.976 | | 7 | 0.362 | | -3.097 – 7.447 | | Control = 45.47±2.197  PSD = 47.64±1.164 |
| Diestrus Phase | 0.159 | | 6 | 0.879 | | -9.247 – 8.117 | | Control = 30.60±3.218  PSD = 30.03±1.496 |
| Estrus Phase | 1.299 | | 6 | 0.242 | | -1.337 – 4.362 | | Control = 27.73±1.121  PSD = 29.24±0.3164 |
| ***GFAP Expression- CA2, Stratum Radiatum*** | | | | | | | | |
| Male, All Subjects | 0.511 | | 8 | 0.623 | | -4.631 – 7.268 | | Control = 42.75±2.982  PSD = 44.06±0.8382 |
| Diestrus Phase | 0.560 | | 6 | 0.596 | | -3.571 – 5.691 | | Control = 28.42±1.788  PSD = 29.48±0.6214 |
| Estrus Phase | 0.900 | | 6 | 0.403 | | -7.950 – 3.675 | | Control = 30.23±0.7722  PSD = 28.09±2.246 |
| ***GFAP Expression- CA1, Pyramidal Layer*** | | | | | | | | |
| Male, All Subjects | 1.013 | | 7 | 0.345 | | -4.348 – 1.287 | | Control = 14.47±0.5033  PSD = 13.17±0.8501 |
| Female, All Subjects | 0.408 | | 14 | 0.690 | | -1.177 – 1.729 | | Control = 9.878±0.5223  PSD = 10.15±0.4315 |
| Diestrus Phase | 0.448 | | 6 | 0.670 | | -2.019 – 2.924 | | Control = 10.09±0.8358  PSD = 10.54±0.5670 |
| Estrus Phase | 0.100 | | 6 | 0.923 | | -2.338 – 2.538 | | Control = 9.668±0.7382  PSD = 9.768±0.6692 |
| ***GFAP Expression- CA2, Pyramidal Layer*** | | | | | | | | |
| Male, All Subjects | 0.981 | | 8 | 0.355 | | -9.280 – 23.03 | | Control = 10.64±0.7007  PSD = 17.52±5.587 |
| Female, All Subjects | 0.045 | | 14 | 0.965 | | -1.217 – 1.167 | | Control = 10.53±0.5117  PSD = 10.50±0.2171 |
| Diestrus Phase | 1.303 | | 6 | 0.240 | | -0.7772 – 2.547 | | Control = 9.903±0.6082  PSD = 10.79±0.3025 |
| Estrus Phase | 1.146 | | 6 | 0.295 | | -2.931 – 1.061 | | Control = 11.15±0.7690  PSD = 10.22±0.2727 |
| **Analysis** | **U** | **p-value** | | | **Median** | | **Mean±SEM** | |
| ***GFAP Expression- CA1, Stratum Radiatum*** | | | | | | | | |
| Female, All Subjects | 20 | 0.234 | | | Control = 28.31, n=8  PSD = 29.42, n=8 | | Control = 29.16±1.668  PSD = 29.64±0.7233 | |
| ***GFAP Expression- CA2, Stratum Radiatum*** | | | | | | | | |
| Female, All Subjects | 28 | 0.721 | | | Control = 29.83, n=8  PSD = 28.90, n=8 | | Control = 29.32±0.9643  PSD = 28.79±1.110 | |

**Table S9.** Statistical analyses for IHC GFAP data.
